# Synthesis and initial screening of lactate dehydrogenase inhibitor activity of 1,3-benzodioxole derivatives

**DOI:** 10.1038/s41598-020-77056-4

**Published:** 2020-11-16

**Authors:** Dicky Annas, Se-Yun Cheon, Mohammad Yusuf, Sung-Jin Bae, Ki-Tae Ha, Kang Hyun Park

**Affiliations:** 1 Department of Chemistry, Pusan National University, Busan, 46241 Republic of Korea; 2 AmcoBio Inc, Seoul, 08758 Republic of Korea; 3 Healthy Aging Korean Medical Research Center and Department of Korean Medical Science, School of Korean Medicine, Pusan National University, Yangsan, 50612 Republic of Korea

**Keywords:** Small molecules, Cancer prevention

## Abstract

Cancer is one of the main causes of mortality in the world. Many cancer cells produce ATP through high-level lactic acid fermentation catalyzed by lactate dehydrogenase (LDH), which converts pyruvic acid to lactic acid. LDH plays a dominant role in the Warburg effect, wherein aerobic glycolysis is favored over oxidative phosphorylation. Due to the high lactic acid production level in cancer cells, LDH-targeting could be a potential cancer treatment strategy. A few approaches, such as drug treatment, reportedly inhibited LDH activity. In this study, we describe new 1,3-benzodioxole derivatives that might be potential small molecule candidates for LDHA inhibition. The synthesis was carried out by trans-esterification between aryl ester and alcohol groups from piperonyl alcohol. Compounds **2** and **10** exhibited a selective LDHA IC_50_ value of 13.63 µM and 47.2 µM, respectively. Whereas only compound **10** showed significant cytotoxicity in several lines of cancer cells, especially in human pancreatic cancer PANC-1 cells. These synthesized compounds possess 2 aromatic rings and –CF_3_ moiety, which expectedly contributes to LDHA inhibition. The presented products have the potential to become a promising LDHA inhibitor drug candidate.

## Introduction

Cancer is one of the biggest health concerns for humans, which takes place at a tissue level^1–3^. Cancer develops through a series of genetic mutations that result in a change in cell fate. The Warburg effect is a phenomenon wherein cancer cells consume more glucose than healthy cells do to ensure ATP supply for energy production and its catabolites as building blocks simultaneously. In particular, ATP and the precursors of lipid, protein, and nucleotide synthesis are produced through glucose conversion during aerobic glycolysis in cancer cells with lactic acid as the primary end product^4^.

Lactate dehydrogenase (LDH) is an enzyme with a tetrameric structure that catalyzes pyruvate conversion to lactate and vice versa. LDH has two known isoforms. LDHA mainly converts pyruvic acid to lactic acid, while LDHB catalyzes the reverse reaction^5^. Several studies have reported that LDHB is constitutively expressed in various cancer cell types, while LDHA is proposedly important for tumor initiation as it is often overexpressed in cancer. Reduced LDHA levels were related to less cellular transformation and delayed tumor formation^6,7^.

**Figure 1:**
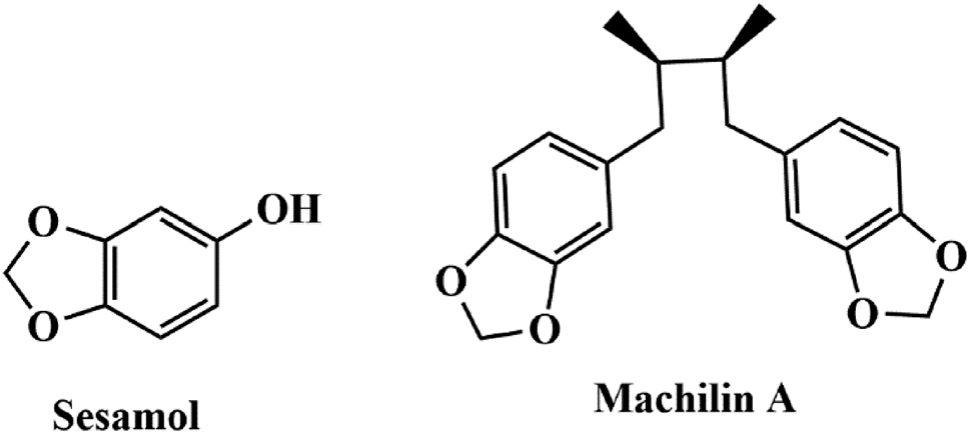
Chemical structure of natural product contained 1,3-benzodioxole rings.

Chemical approaches are among the common strategies to inhibit LDHA activity in cancer cells. The 1,3-benzodioxole ring was described as a component of many natural compounds with various biological activities. These compounds and their derivatives are widely-used pesticides and herbicides^8^. Certain studies reported that the 1,3-benzodioxole ring possesses antitumor, antiparasitic, antifungal, antioxidant, and antibacterial bioactivities^9–13^, such as the antioxidant sesamol (Fig. 1)^14^. In addition, 1,3-benzodioxole derivatives could act as carcinogenesis-associated histone deacetylase inhibitors during cancer treatment^15^.

Several 1,3-benzodioxole ring-containing chemical compounds have been developed with the aim to inhibit LDHA activity. These compounds include 1,3-benzodioxole derivatives, such as Machilin A (Fig. 1), which are efficient competitive inhibitors that function by blocking the nicotinamide adenine dinucleotide (NAD) binding site of LDHA, suppressing lactate production and cancer cell growth^16^. Furthermore, benzodioxole ring-containing thiazolyl-pyrazoline derivatives were tested against MCF-7 and B16-F10 tumor cells and showed significant antiproliferative activity in vitro^17^. In addition, we previously reported that a selenobenzene compound harboring trifluoromethyl group, 1-(phenylseleno)-4-(trifluoromethyl) benzene, showed an anti-tumor effect thorough suppressing LDHA activity^18^. Based on the aforementioned results, we developed new 1,3-benzodioxole and trifluoromethyl derivatives through trans-esterification and evaluated the in vitro LDHA inhibitor activity of the synthesized compounds through decreasing NADH intensity.

## Results and discussion

The main chemical reaction in this study was a trans-esterification reaction between the ester group of ethyl 4-bromobenzoate (**1a**) and the alcohol groups of piperonyl alcohol (**1b**). A base was used to abstract the protons from the alcohol groups, creating an anion that could directly abstract the carbonyl in the ester groups, and release the alcohol moiety^19^. The reaction between ethyl 4-bromobenzoate and piperonyl alcohol was carried out as presented in Table 1. Initially, the study was carried out using pyridine as a base in toluene at 110 °C under air condition and the reaction mixture was stirred for 12 h. The yield of the product was 39% (Table 1, entry 1). The reaction using K_2_CO_3_ as a base resulted in a lower yield compared to that using pyridine, which yielded 32% (Table 1, entry 2). By increasing the degree of basicity, the product could be obtained with yields of 45% and 53% in the case of NaOH and KOH, respectively (Table 1, entry 3–4). The highest yield (74%) was obtained when Cs_2_CO_3_ was used as a base under these reaction conditions (Table 1, entry 5). Therefore, Cs_2_CO_3_ was selected as a reaction base for further optimization due to its higher activity, yield, and catalytic speed compared to acid catalysts^20^. The transesterification mechanism using Cs_2_CO_3_ has proposed^21^. The carbonyl group coordinates with a metal ion to make the carbon center more electrophilic, while the alcohol group is activated by carbonate ion to make a negative charge on the oxygen of the hydroxyl group. This anion directly abstracts the activated carbonyl to form the ester group and release ethanol.

**Table 1:** Optimization of reaction conditions for synthesis of compound 1 to 8.


Entry	Base	Solvent	T (°C)	t (h)	Yield^**a**^ (%)
1	Pyridine	Toluene	110	12	39
2	K_2_CO_3_	Toluene	110	12	32
3	NaOH	Toluene	110	12	45
4	KOH	Toluene	110	12	53
5	Cs_2_CO_3_	Toluene	110	12	74
6	Cs_2_CO_3_	DMSO	110	12	40
7	Cs_2_CO_3_	DCM	40	12	Trace
8	Cs_2_CO_3_	THF	60	12	20
9	Cs_2_CO_3_	Toluene	25	12	Trace
10	Cs_2_CO_3_	Toluene	60	12	29
11	Cs_2_CO_3_	Toluene	110	18	91
12	Cs_2_CO_3_	Toluene	110	24	94

Reaction conditions: ethyl 4-bromobenzoate (1 mmol), piperonyl alcohol (1.1 mmol), Cs_2_CO_3_ (1 mmol), and toluene (5 mL).

^a^Isolated yields.

We then screened for the solvent effect in this reaction. When an aprotic polar solvent, such as DMSO, was used, the product yield decreased to 40% (Table 1, entry 6). No reaction could be observed when DCM was used as a solvent (Table 1, entry 7), whereas reaction using THF was less successful (Table 1, entry 8). We could conclude that toluene was the best solvent for this reaction. The solvent effect plays an important role in organic equilibrium reactions, such as tautomerization, electron transfer reaction, isomerization, and acid–base balance^22^.

Furthermore, the reaction was tested at different conduction temperatures. At room temperature, no product was observed (Table 1, entry 9). We could only detect a yield of 29% at 60 °C (Table 1, entry 10). We also aimed at optimizing the reaction time. When the reaction time was increased to 18 h, an excellent yield (91%) was obtained (Table 1, entry 11). We observed no significant difference when the reaction time was increased to 24 h (Table 1, entry 12). Based on the optimization results, we selected Cs_2_CO_3_ as a base, toluene as a solvent, 110 °C as reaction temperature, and 18 h as the reaction time for further experiments.

**Figure 2:**
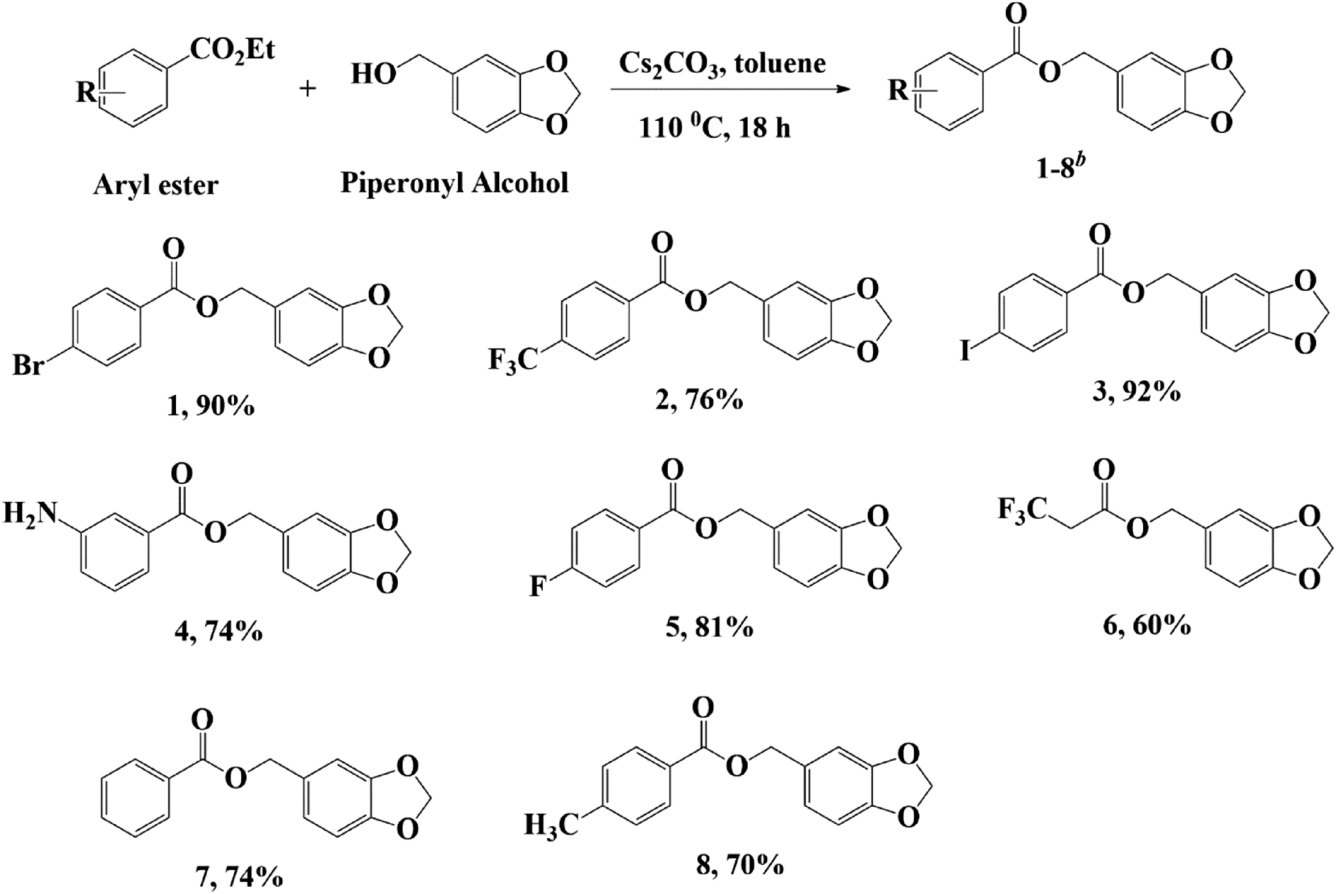
Substrate scope. Reaction conditions: Arylethyl ester (1 mmol), piperonyl alcohol (1.1 mmol), Cs_2_CO_3_ (1 mmol), and toluene (5 mL). ^b^Isolated yields.

Under these optimized conditions, we synthesized other 1,3-benzodioxole derivatives by changing the R substituent. This method can tolerant some of the substituents in good to excellent yield (Fig. 2). Aryl ester as a substrate with halogen substituents (*p*-Br, *p*-I, and *p*-F) resulted in excellent yield, between approximately 81 and 92% (compound **1**, **3**, and** 5**). Furthermore, reactions with trifluoromethyl (*p*-CF_3_) and amine (*m*-NH_2_) as substituents also proceeded smoothly and resulted in the products **2** and **4**, with a yield of 76% and 74%, respectively. The addition of electron-donating groups, such as in the case of compounds **7** and **8**, also resulted in good yields of 74% and 70%, respectively. However, the use of –CF_3_ as a substituent did not provide a favorable result when it was not attached to the aromatic ring (compound **5**). This suggested that the aromatic ring played a role in the reaction efficiency as it could delocalize electrons that would result in more electrophilic carbonyl groups.

**Figure 3:**
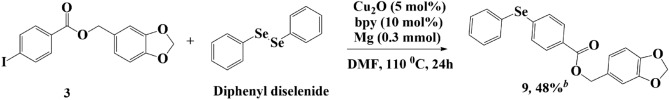
Synthesis of compound 9. Reaction conditions: Compound **3c** (0.6 mmol), diphenyl diselenide (0.3 mmol), and DMF (1 mL). ^b^Isolated yields.

Selenide compounds reportedly exhibit various bioactivities, such as antioxidant, antibacterial, and anticancer effects^23–25^. In our study, we also developed 1,3-benzodioxole-modified selenide compounds as we expected that such modifications would further increase the bioactivity of the compound. The modification of the selenide compound and 1,3-benzodioxole was achieved by reacting aryl iodide with diphenyl diselenide via the cleavage of the Se–Se bond (Fig. 3). Our results showed that compound **3**, the product of the trans-esterification from the previous reaction, acted as an aryl iodide compound that reacted with diphenyl diselenide to form an asymmetrical diaryl chalcogenide compound. Using this method, we successfully synthesized target product **9** with a 48% yield.

**Figure 4:**
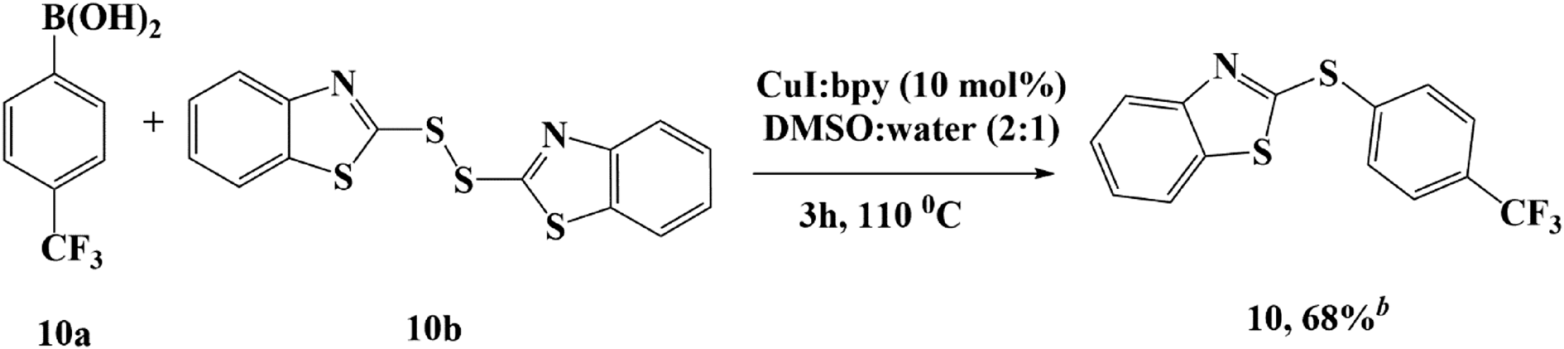
Synthesis of compound 10. Reaction conditions: phenyl boronic acid (1.3 mmol), 2,2-dithiobis(benzothiazole) (0.6 mmol), and DMSO:water (2:1). ^b^Isolated yields.

Apart from the selenide compound modification, we also synthesized an aryl-heteroatom C–S bond, with a heterocyclic group in order to study its bioactivity as an LDHA inhibitor. This compound was used as a comparison for a 1,3-benzodioxole ring and *p*-CF_3_ moiety in the structure of compound **2**. N and S heterocyclic compounds are well-known for their diverse biological activities and are used in several diseases treatments^26–28^. In our study, we synthesized such a compound using (trifluoromethyl)phenylboronic acid and 2,2′-dithiobis(benzothiazole) via S–S cleavage, resulting in a C–S bond with an N and S heterocyclic ring **10**, with a 68% yield (Fig. 4).

The in vitro evaluation of the LDHA inhibitor activities of the synthesized compounds was determined by the NADH intensity decrease through oxidation in a solution of HEPES-K^+^, NADH, and pyruvate at a pH = 7.2. The NADH oxidation fluorescence intensity was measured using a spectrofluorometer at 340 nm excitation and 460 nm emission wavelengths, representing the NADH-specific fluorescence spectrum. Furthermore, the in vitro evaluation of the LDHB inhibitor activity was determined as a reverse reaction, which converts lactate to pyruvate by determining the amount of NAD^+^ converted to NADH using the above-mentioned spectrophotometric experimental setting (Table 2). Based on this analysis, the half-maximal inhibitory concentration (IC_50_) of the synthesized compounds was obtained.

**Table 2:** Bioactivity of synthesized compounds in inhibits LDHA and LDHB.

Entry	Compounds	LDHA IC_50_	LDHB IC_50_	LDHB/LDHA
1	1	> 1000 µM	79.05 µM	ND
2	2	13.63 µM	395.3 µM	29.00
3	3	> 1000 µM	150.8 µM	ND
4	4	182.5 µM	7.87 µM	0.043
5	5	477.5 µM	> 1000 µM	2.094
6	6	452.5 µM	129.8 µM	0.29
7	7	842.6 µM	> 1000 µM	> 1.18
8	8	> 1000 µM	248 µM	ND
9	9	> 1000 µM	151.1 µM	ND
10	10	47.20 µM	> 1000 µM	> 21.18
11	GSK2837808A	2.6 nM	130.3 nM	50.12
12	GNE140	59.9 nM	151.1 nM	2.52

*ND* not determined.

We used GSK2837808A and GNE140 as standard compounds in this measurement that is a routinely used potential LDH inhibitor with an IC_50_ of 2.6 nM and 59.9 nM, respectively. Since LDHA is often overexpressed in cancer, LDHB was used to evaluate the LDHA-selectivity of the synthesized compounds. We tested various aryl esters, presented in Fig. 2, of which compound **1** (*p*-Br), **3** (*p*-I), and **8** (*p*-CH_3_) exhibited the highest LDHA IC_50_ values of over 1000 µM. However, compound **1** exhibited the lowest LDHB IC_50_ value of 79.05 µM, indicating that compound **1** is a selective LDHB inhibitor compare to the other 3 compounds. Moreover, compound **7** also showed high LDHA IC_50_ value (842.6 µM) and LDHB IC_50_ value (over 1000 µM) values. These values were too high to be compared with the standards; thus, we concluded that these 4 aryl ester compounds were inactive LDHA inhibitors.

Furthermore, compound **5**, with 2 aromatic rings and a *p*-F moiety, exhibited a moderate LDHA IC_50_ value of 477.5 µM and was a selective LDHA inhibitor due to its LDHB IC_50_ value, which was greater than 1000 µM. Besides compound **5**, compound **6**, with only one aromatic ring and a –CF_3_ moiety, also had a moderate LDHA IC_50_ value of 452.5 µM. However, compound **6** was not selective for LDHA based on its LDHB IC_50_ value, which was lower than its LDHA IC_50_ value. In addition, compound **4**, containing an *m*-NH_2_ moiety, was not selective for LDHA either, as we measured the lowest LDHB IC_50_ value (7.87 µM) in the case of this compound. The lowest and selective LDHA inhibitor was compound **2** (13.63 µM) with 2 aromatic rings and *p*-CF_3_ moiety. This compound was selective as LDHA inhibitor compares to its LDHB inhibitor activity (LDHB IC_50_ value of 395.3 µM). These values were still higher compared to the IC_50_ values of the GSK2837808A and GNE140 standards but lower compared to other established LDHA inhibitors, such as galloflavin, which was also used as a reference compound since it is routinely used in enzymatic LDH assays, with an IC_50_ value of approximately 110 µM^29^. This result suggested that it could potentially become LDHA inhibitor drug candidate. Moreover, we also synthesized selenide compounds, containing 1,3-benzodioxole (**9**) and benzothiazole (**10**) groups. Compound **9** was not efficient with LDHA and LDHB IC_50_ values of 1000 µM, while compound **10** exhibited a favorable selectivity as an LDHA inhibitor with an LDHA IC_50_ value of 47.20 µM and an LDHB IC_50_ value of over 1000 µM.

To assess the anticancer potency of these compounds in several cancer cells, we determined the cell toxicities of these compounds using an MTT assay. As shown in Table 3, the compound **2** showed was too weak. It was over 1000 µM of the concentration of half-maximal growth inhibition (GI_50_) value. On the other hand, compound **10** has higher cytotoxicity compared to other compounds in various cancer cell lines. Among these cell lines, compound **10** is most effective in reducing the cell viability in human pancreatic cancer PANC-1 cells (GI_50_ value of 12.19 µM). In addition, we analyzed the cytotoxicity of two standard compounds, GSK2837808A and GNE140. The calculated GI_50_ values of GSK2837808A and GNE140 were 11.31 µM and 11.59 µM, respectively. There is a small difference between standard compounds and compound **10** in growth inhibition of PANC-1 cells.

**Table 3:** The 50% growth inhibition concentration (GI_50_) of compounds on various human cancer cell lines.

Entry	Compounds	Cell lines				
		PANC-1 (GI_50_) (µM)	A549 (GI_50_) (µM)	MCF-7 (GI_50_) (µM)	MiaPaCa-2 (GI_50_) (µM)	U87 (GI_50_) (µM)
1	1	> 1000	> 1000	> 1000	> 1000	> 1000
2	2	> 1000	> 1000	> 1000	> 1000	> 1000
3	3	269.3	> 1000	> 1000	> 1000	> 1000
4	4	203.4	> 1000	729	> 1000	379.5
5	5	643.9	> 1000	> 1000	> 1000	> 1000
6	6	> 1000	> 1000	> 1000	> 1000	> 1000
7	7	243.3	> 1000	> 1000	> 1000	> 1000
8	8	86.46	> 1000	> 1000	> 1000	> 1000
9	9	21.11	924.8	455.1	108	> 1000
10	10	12.19	56.46	61.38	88.88	343.4
	GSK2837808A	11.31	ND	ND	ND	ND
	GNE140	11.93	ND	ND	ND	ND

*ND* not determined.

In addition to intracellular anticancer activity, to provide the basic in vitro drug-like data of compound **10**, we performed basically in vitro assays, such as human liver microsomal stability, plasma stability, and CYP inhibition (Table 4). The compound **10** was rapidly metabolized by human and rat microsomes; only 9.5% and 5.2% of compound **10** remained after 30 min of incubation. However, a plasma stability test showed compound **10** was stable in both human and rat plasma. The compound **10** is a moderate perpetrator of drug–drug interactions based on their inhibition of the most abundant CYP450 enzymes, such as 1A2 and 2C19.

**Table 4:** Summary of in vitro ADME for compound **10**.

Compounds	CYP1A2	CYP2C9	CYP2C19	CYP2D6	CYP3A4
**CYP isozyme activity (% of control activity)**					
Compound 10	36.7	97.4	12.1	95.1	> 100
Ketoconazole (reference)	99.1	97.5	> 100	99.0	25.0
Ketoconazole: CYP3A4 inhibitor (0.1 µM)					

Compounds	Human (%)		Rat (%)		
**Human and rat liver microsomal stability (% remaining during 30 min)**					
Compound 10	9.5		5.2		
Verapamil (reference)	15.3		–		

Compounds	Human		Rat		
	30 min	120 min	30 min	120 min	
**Human and rat plasma stability (% remaining)**					
Compound 10	98.1	91.6	93.8	87.5	
Procaine (reference)	1.5 (5 min)	0.4 (10 min)	89.8	52.6	
Enalapril (reference)	95.0	93.7	37.7	1.8	

The compound **2** exhibits a simple structure with one 1,3-benzodioxole group compared to the previously reported Machilin A^16^, containing two 1,3-benzodioxole groups. Furthermore, it has a lower LDHA IC_50_ value compared to Machilin A (84 µM). Sada et al*.*, also presented the LDHA inhibitory activity of stiripentol analogs at a higher dose (500 µM)^30^. However, piperonyl alcohol and 1,3-benzodioxole did not exhibit an LDHA inhibitory activity even at concentrations up to 1 mM^16^. However, unlike the previously reported compounds harboring 1,3-benzodioxole group, compound **2** did has an anti-cancer effect in several lines of cancer cells. It could be a result of its chemical properties including low stability or poor cellular uptake. Compound **10**, a benzothiazole compound harboring a –CF_3_ moiety, could also be considered as a potential LDHA inhibitor. Although higher IC_50_ in vitro LDHA assay, the GI_50_ value of compound **10** is lower than that of previously reported selenobenzene compound, 1-(phenylseleno)-4-(trifluoromethyl) benzene^18^. The GI_50_ value of compound **10** in PANC-1 cells is comparable to that of standard compounds, GSK2837808A and GNE140.

From these results, compound **2** and **10** among the synthesized compounds, with the simple structure and comparable activity, could be potentially used as an LDHA inhibitor and should be further investigated. These synthesized compounds possess 2 aromatic rings and –CF_3_ moiety, which is expected to contribute to LDHA inhibition. The compounds have the potential to become a promising LDHA inhibitor for the anticancer drug candidate. To improve the in vitro LDHA inhibition and intracellular activity of these compounds, it is needed to conduct an extensive structure–activity relationship study, including substitutions in a different position, bioisosteres replacement, and scaffold hopping.

## Conclusions

Here we described 1,3-benzodioxole derivatives, synthesized through trans-esterification between aryl ester and piperonyl alcohol groups. Through this approach, the substrate scope of this reaction was also investigated and could tolerate many substituents, with good to excellent yields. In addition, we also synthesized benzothiazole derivatives and 1,3-benzodioxole-modified selenide compounds. Compound **2**, containing a benzodioxole ring and a –CF_3_ moiety, showed a potent inhibitory action in vitro assay, however, failed to show anticancer effect in human cancer cells. Compound **10**, a benzothiazole harboring a –CF_3_ group, showed both activities of in vitro LDHA inhibition and intracellular cytotoxicity. These compounds could potentially be used as an LDHA inhibitor due to its optimal activity and selectivity based on the decrease in the NADH intensity and as it has the smallest IC_50_ among all the compounds. Thus, compound **10** could be considered a potent LDHA inhibitor for further in vivo evaluations.

## Methods

### General methods

All chemicals and reagents were purchased from Tokyo Chemical Industry (Tokyo, Japan) and Sigma-Aldrich (St. Louis, MO) and used without further purification. Fourier-transform infrared (FT-IR) spectra were recorded on NICOLET 380. ^1^H (400 MHz) and ^13^C (100 MHz) NMR of all synthesized compounds were recorded on Bruker Magnet System 400′54 Ascend. Gas Chromatography–Mass Spectrometry spectra were performed on Shimadzu GC-1010 Plus GCMS-QP2010 SE. High-resolution Mass Spectrometry (HR-MS) data were performed on 6530Accurate-Mass Q-TOF LC/MS. Spectrofluorometer spectra were performed on Spectramax M2; Molecular Devices, Sunnyvale, CA, USA.

### Synthesis of compound 1–8

Arylethyl ester (1 mmol) was reacted with piperonyl alcohol (1.1 mmol) in 5 mL toluene. Cesium carbonate (1 mmol) was added to the mixture as a base. The reaction mixture was stirred at reflux under air condition for 18 h. The reaction was monitored by TLC and GC–MS for completion reaction. After the completion reaction, the mixture was cooled to room temperature. The mixture was diluted by dichloromethane and evaporate the solvent. The crude product was stored in a refrigerator for 24 h to conduct white solid. The crude product was purified by column chromatography over silica gel.

### Synthesis of compound 9

The reactions referred to^31^ with modification. Compound **3** (0.6 mmol) was reacted with diphenyl diselenide (0.3 mmol) in 1 mL DMF. Cu_2_O (5 mol%), bpy (10 mol%), and Mg (0.6 mmol) were added to the reaction mixture. The mixture was stirred at 110 °C for 24 h. The reaction was monitored by TLC and GC–MS. After the completion reaction, the mixture was cooled to room temperature. Then, the crude product was separated by an extraction process using dichloromethane and brine solution. The organic layer was evaporated and the crude product was purified by column chromatography over silica gel.

### Synthesis of compound 10

The reactions referred to^32^ with modification. Trifluoromethyl phenyl boronic acid **10a** (1.3 mmol) was reacted with 2,2-dithiobis(benzothiazole) **10b** (0.6 mmol) in DMSO:water (2:1). CuI;bpy (10 mol%) was added to the reaction mixture. The mixture was stirred at 110 °C for 3 h. The reaction was monitored by TLC and GC–MS. After the completion reaction, the mixture was cooled to room temperature. Then, the crude product was separated by an extraction process using dichloromethane and brine solution. The organic layer was evaporated and the crude product was purified by column chromatography over silica gel.

In vitro evaluation on LDHA inhibitor activity**.** LDHA activity assay was performed in accordance with previous studies^16,18^. Briefly, the various concentrations of compounds were incubated with reaction buffer containing 20 mM HEPES-K^+^, 20 μM NADH, 2 mM pyruvate, and 100 ng of purified recombinant human LDHA protein (Abcam, Cambridge, UK). The fluorescence of NADH, which has an excitation wavelength of 340 nm and an emission wavelength of 460 nm, was detected using a microplate spectrofluorometer (Spectramax M2; Molecular Devices, Sunnyvale, CA).

### In vitro LDHB Activity Assay

In vitro analysis was determined the amount of NAD^+^ converted to NADH^33^. The assay mixture composed of 100 mM Tris–HCl buffer (pH 8.0), 200 mM sodium L-lactate, 2.5 mM NAD^+^, and 100 ng of purified recombinant human LDHB protein (Abcam). The fluorescence intensity of NADH was measured using a spectrofluorometer (Spectramax M2) at 340 nm as excitation wavelength and 460 nm as emission wavelength which is the specific fluorescence of NADH.

### Cell culture

The human pancreatic cancer cell lines, PANC-1 and MiaPaCa-2, human lung cancer A549 cells, human breast cancer MCF-7, and human glioma U87 cells were obtained from the Korean cell line bank (Seoul, Korea). The cells were cultured in Dulbecco’s Modified Eagle Medium (DMEM; ThermoFisher Scientific, Waltham, MA) supplemented with 10% fetal bovine serum (FBS; Sigma-Aldrich) and 1% penicillin/streptomycin (ThermoFisher Scientific). The cells were cultured at 37 °C in an atmosphere containing 5% CO_2_.

### Intracellular LDHA activity assay

To observe the LDH activities from the lysates of cells, we performed in accordance with previous studies^16,18^. Briefly, Total protein from lysate (1 μg) was mixed with containing 20 mM HEPES-K^+^, 20 μM NADH, 2 mM pyruvate. The fluorescence of NADH, which has an excitation wavelength of 340 nm and an emission wavelength of 460 nm, was detected using a microplate spectrofluorometer (Spectramax M2).

### Cell viability

The potential cytotoxicity of compounds at different concentrations was evaluated using the MTT assay. Briefly, cancer cell lines were pre-incubated in 96-well plates with compounds for 48 h. Subsequently, MTT working solution (2 mg/mL in phosphate buffer solution) was added to each well and the plate was incubated for 4 h at 37 °C in an atmosphere containing 5% CO_2_. Then, the conditioned media were aspirated, and the formed formazan crystals in living cells were quantified using the microplate reader (Spectramax M2) at 540 nm. The concentrations that produce 50% cell growth inhibition (GI_50_) were calculated by curves constructed by the plot of cell survival by an assistant of PRISM software (GraphPad, San Diego, CA).

### LC–MS/MS analysis

Chromatographic separation was performed on the Shimadzu Nexera XR system (Kyoto, Japan). Detection was performed on a Thermo TSQ Vantage triple quadrupole LC–MS/MS (MA, USA). The analytes were separated on a Phenomenex Kinetex C18 (2.1 × 100 mm, 2.6 µm particle size) column (Torrance, USA). The mobile phase system consisted of water containing 0.1% formic acid (mobile phase A, MPA), and acetonitrile containing 0.1% formic acid (mobile phase B, MPB). Xcalibur 1.6.1 software (Thermo Scientific) was used for data acquisition and processing.

### Plasma stability

Human and rat plasma (Sigma-Aldrich) are incubated at 37 °C with test compounds. During the incubation, aliquots are withdrawn at 0, 30, 120 min time points and acetonitrile solution (containing chlorpropamide) is added. After vortexing, the aliquots are centrifuged and the supernatant is withdrawn for analysis by LC–MS/MS.

### CYP isozymes activity assay

Pooled Human liver microsomes (Sigma-Aldrich; 0.25 mg/mL), 0.1 M phosphate buffer solution (pH 7.4), the five most commonly used substrate cocktails, such as 50 µM phenacetin (CYP1A2), 10 µM diclofenac (CYP), 100 µM S-mephenytoin (CYP2C19) 5 µM dextromethorphan (CYP2D6), and 2.5 µM midazolam (CYP3A4), and compound 10 are pre-incubated at 37 °C for 5 min, then incubated with NADPH generation system solution for 15 min. To finish the enzymatic reaction, an acetonitrile solution (containing terfenadine) is added. The reaction tubes are centrifuged and the supernatant is withdrawn for analysis by LC–MS/MS.

### Microsomal stability

The assay use liver microsomes from two species (human, rat, 0.5 mg/mL). liver microsomes preincubated with 0.1 M PBS (pH 7.4) and 1 µM compound 10 at 37 °C for 5 min, then incubated with NADPH regeneration system solution for 30 min. To finish the reaction, an acetonitrile solution (involved in chlorpropamide) is added. The reaction tubes are centrifuged and the supernatant is withdrawn for analysis by LC–MS/MS.

### Statistical analysis

Results were presented as mean ± standard deviation (SD) of triplicate experiments. Statistical analysis was performed using one-way ANOVA followed by Dunnett’s post hoc test, and *p* values of lesser than 0.05 were considered statistically significant.

## Supplementary information

Supplementary information.

## Data Availability

All data generated or analyzed during this study are included in this published article (and its Supplementary Information Files).
