# Supplementary material for: Synthesis and initial screening of lactate dehydrogenase inhibitor activity of 1,3-benzodioxole derivatives
Source: Sci Rep. 2020 Nov 16;10:19889. doi: 10.1038/s41598-020-77056-4 (PMC7669867; doi:10.1038/s41598-020-77056-4)
Supplement: Supplementary file 1 — Supplementary information. [file 41598_2020_77056_MOESM1_ESM.pdf]

Supplementary information

Synthesis and initial screening of lactate dehydrogenase inhibitor activity of 1,3-benzodioxole derivatives

Dicky Annas†[1], Se-Yun Cheon†[2], Mohammad Yusuf[1], Sung-Jin Bae[3], Ki-Tae Ha[3,*] and Kang Hyun Park[1,*]

[1] Department of Chemistry, Pusan National University, Busan, 46241, Republic of Korea

[2] AmcoBio Inc., Seoul, 08758, Republic of Korea

[3] Healthy Aging Korean Medical Research Center and Department of Korean Medical Science, School of Korean Medicine, Pusan National University, Yangsan, 50612, Republic of Korea

[*]Correspondence to: hagis@pusan.ac.kr (K.T.H.), chemistry@pusan.ac.kr (K.H.P.)

† These authors contributed equally to this work.

Synthesis of compound 1-8. Arylethyl ester (1 mmol) was reacted with piperonyl alcohol (1.1 mmol) in 5 mL toluene. Cesium carbonate (1 mmol) was added to the mixture as a base. The reaction mixture was stirred at reflux under air condition for 18 hours. The reaction was monitored by TLC and GC-MS for completion reaction. After the completion reaction, the mixture was cooled to room temperature. The mixture was diluted by dichloromethane and evaporate the solvent. The crude product was stored in refrigerator for 24 hours to conduct white solid. Crude product was purified by column chromatography over silica gel.

***Benzo[d][1,3]dioxol-5-ylmethyl 4-bromobenzoate* (1):** white solid; IR (KBr) (ν_{max}/cm^{-1}): 1713, 1498, 1441, 1260, and 774 cm^{-1}. ^{1}H NMR (400 MHz, $CDCl_3$). δ = 5.17 (s, 2 H), 5.91 (s, 2 H), 6.72-6.73 (d, 1 H), 6.83 (d, 1 H), 6.86 (s, 1 H), 7.49-7.51 (d, 2 H), 7.83-7.85 (d, 2 H). ^{13}C NMR (100 MHz, $CDCl_3$): δ = 67, 101.25, 108.25, 109.09, 122.39, 129.5, 131.23, 131.73, 147.89, 165.70. MS (ESI): m/z calcd for $C_{15}H_{11}BrO_4$+Na:356.97; found: 356.9737 [M+Na].

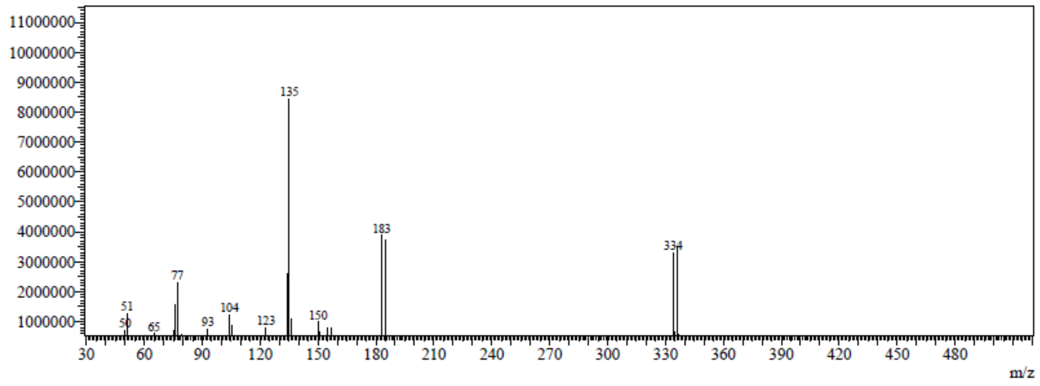

Figure S1. GC-MS spectrum of compound 1

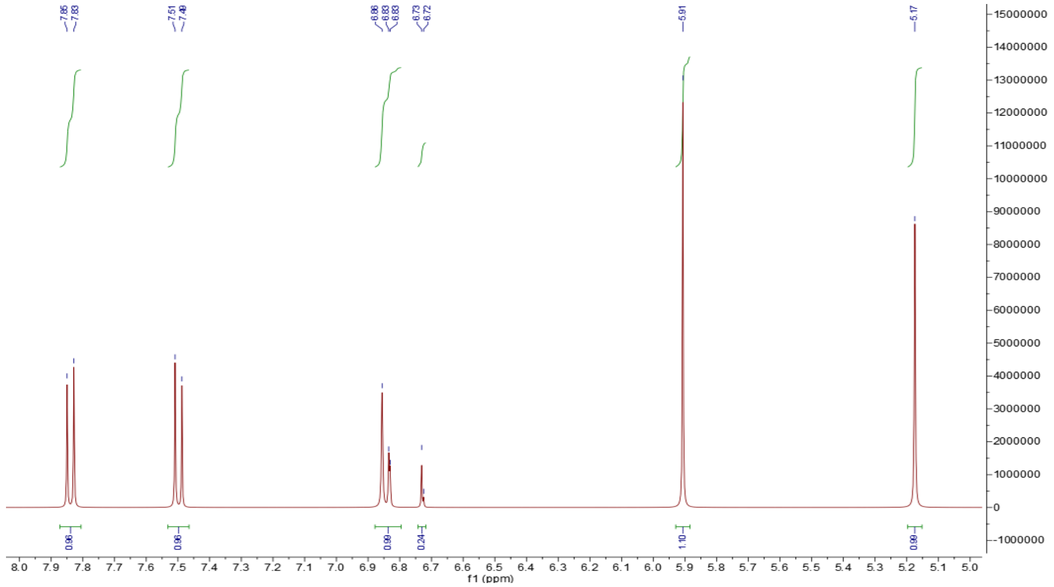

Figure S2. ^{1}H NMR spectrum of compound 1

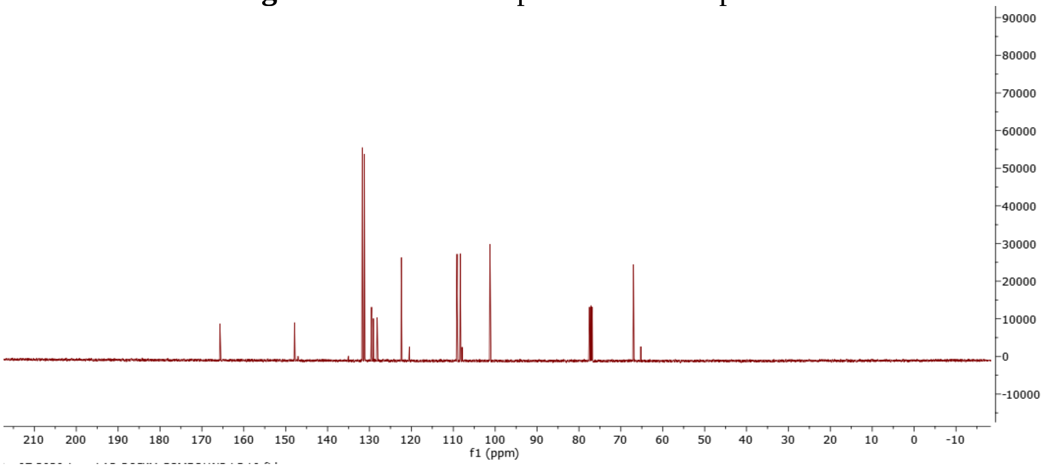

Figure S3. ^{13}C NMR spectrum of compound 1

Benzo[d][1,3]dioxol-5-ylmethyl 4-(trifluoromethyl)benzoate **(2):** white solid; IR (KBr) (ν_{max}/cm^{-1}): 1710, 1489, 1441, 1262, and 1325 cm^{-1}. ^{1}H NMR (400 MHz, $CDCl_3$). δ = 5.21 (s, 2 H), 5.91 (s, 2 H), 6.74-6.76 (d, 1 H), 6.85 (d, 1 H), 6.87 (s, 1 H), 7.62-7.64 (d, 2 H), 8.10-8.11 (d, 2 H). ^{13}C NMR (100 MHz, $CDCl_3$): δ = 67.28, 101.2, 108.11, 109.30, 122.20, 125.03, 129.20, 130.17, 133.45,134.42, 135.32, 147.06, 147.92, 165.25. MS (ESI): m/z calcd for $C_{16}H_{11}F_3O_4$+Na:347.05; found: 347.0638 [M+Na].

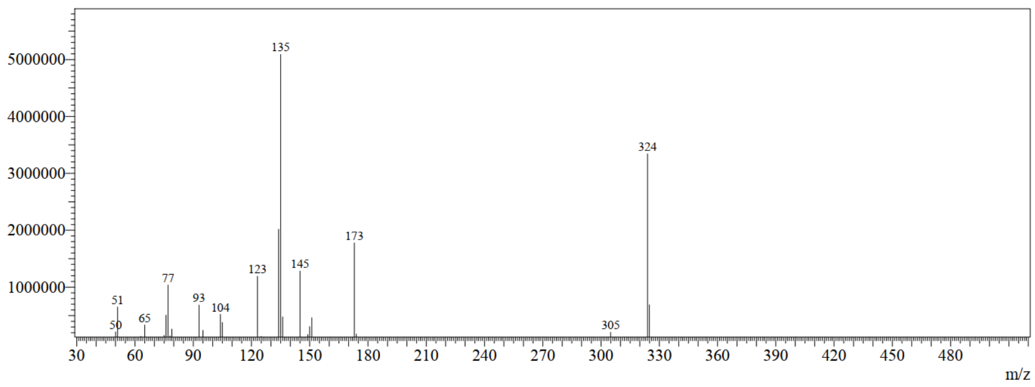

Figure S4. GC-MS spectrum of compound 2

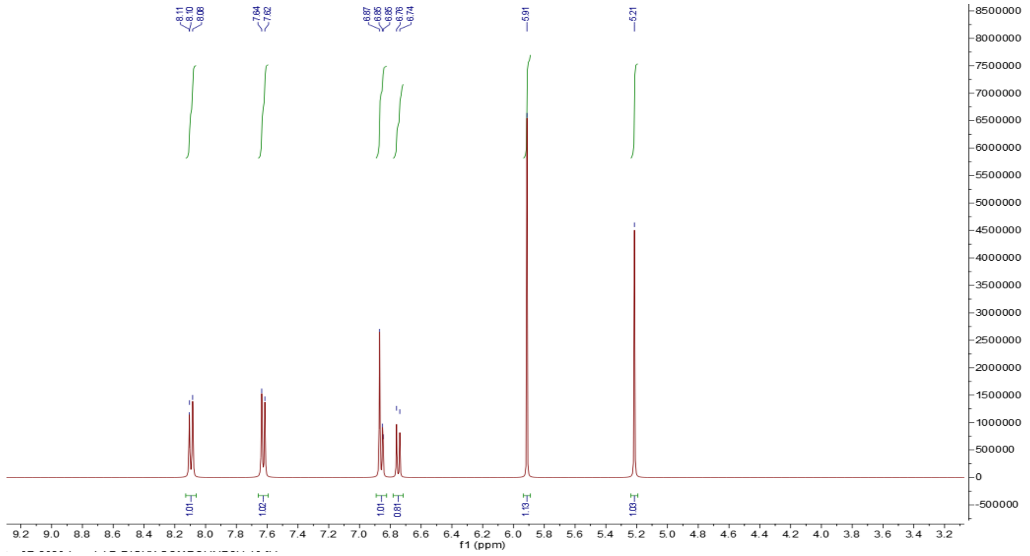

Figure S5. ^{1}H NMR spectrum of compound 2

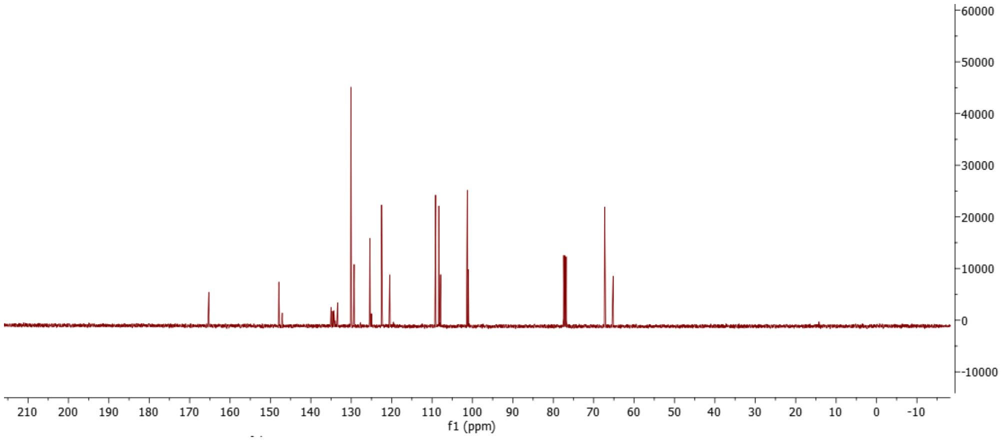

Figure S6. ^{13}C NMR spectrum of compound 2

***Benzo[d][1,3]dioxol-5-ylmethyl 4-iodobenzoate* (3):** white solid; IR (KBr) (ν_{max}/cm^{-1}): 1710, 1495, 1439, 1239, and 682 cm^{-1}. ^1H NMR (400 MHz, $CDCl_3$). δ = 5.17 (s, 2 H), 5.91 (s, 2 H), 6.73-6.75 (d, 1 H), 6.83 (d, 1 H), 6.85 (s, 1 H), 7.67-7.69 (d, 2 H), 7.71-7.73 (d, 2 H). ^{13}C NMR (100 MHz, $CDCl_3$): δ = 66.88, 101.25, 108.33, 109.10, 122.40, 129.50, 129.63, 131.14, 137.74, 117.88, 165.94. MS (ESI): m/z calcd for $C_{15}H_{11}IO_4$+Na:404.96; found: 403.9737 [M+Na].

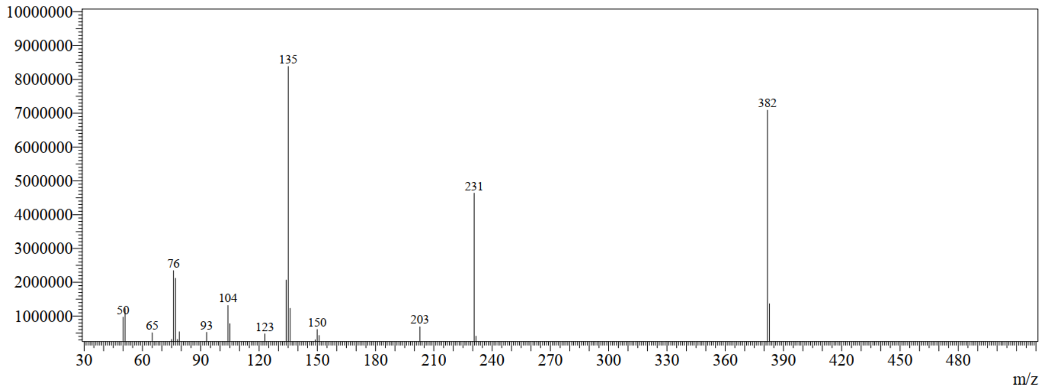

Figure S7. GC-MS spectrum of compound 3

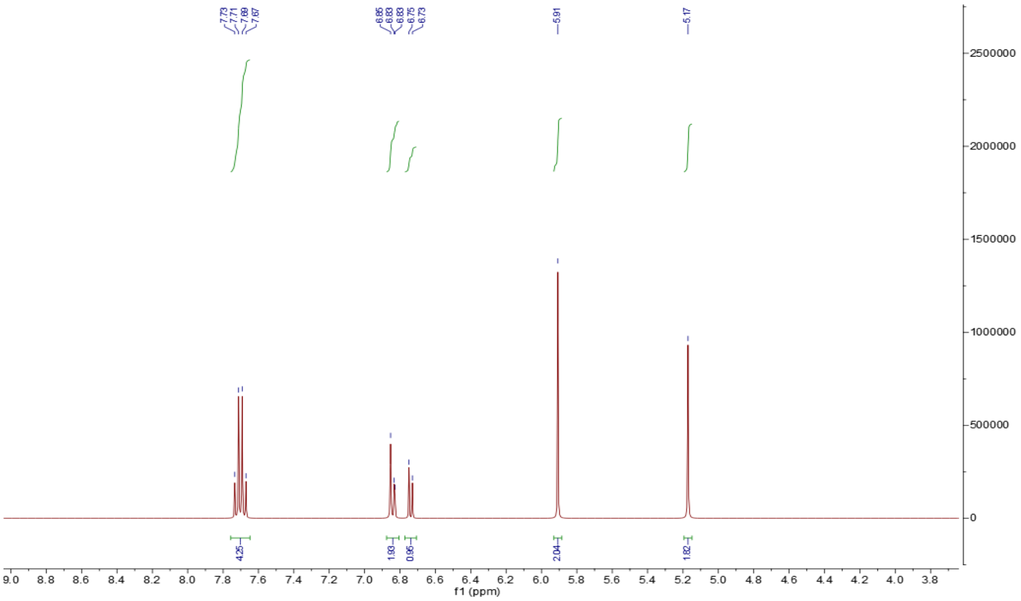

Figure S8. ^1H NMR spectrum of compound 3

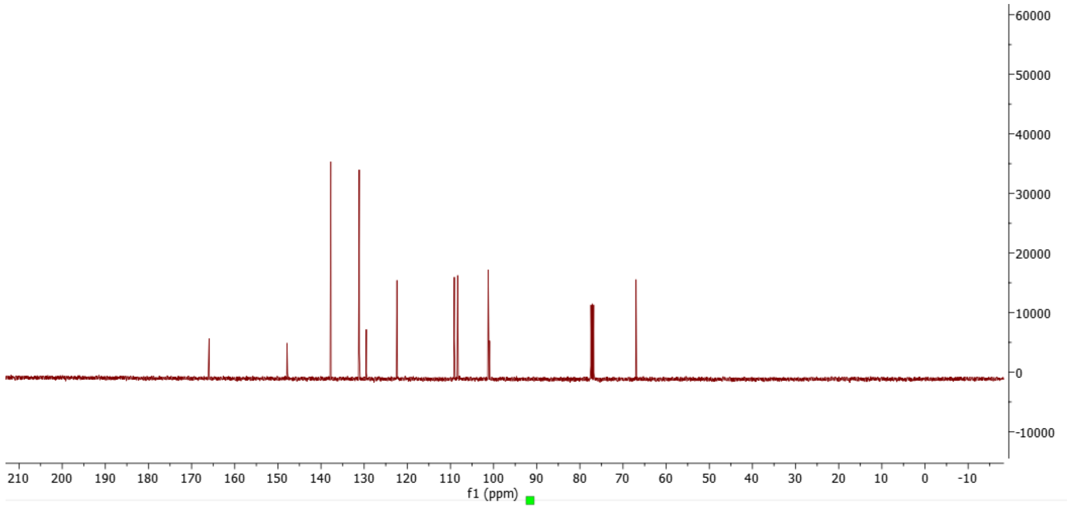

Figure S9. ^{13}C NMR spectrum of compound 3

Benzo[d][1,3]dioxol-5-ylmethyl 3-aminobenzoate **(4):** brown solid; IR (KBr) (ν_{max}/cm^{-1}): 3372, 1669, 1490, 1442, and 1263 cm^{-1}. ^{1}H NMR (400 MHz, $CDCl_3$). δ = 5.16 (s, 2 H), 5.19 (s, 2 H), 5.88 (s, 2 H), 6.71-6.72 (d, 1 H), 6.80-6.81 (d, 1 H), 6.82-6.83 (d, 1 H), 6.85 (s, 1H), 7.20-7.24 (dd, 1 H), 7.25 (s, 1 H), 7.67-7.68 (d, 1H). ^{13}C NMR (100 MHz, $CDCl_3$): δ = 66.65, 101.1, 115.91, 119.49, 119.66, 119.88, 122.20, 129.27, 129.32, 130.84, 146.36, 147.01, 147.83, 166.68. MS (ESI): m/z calcd for $C_{15}H_{13}NO_4$+H:272.09; found: 272.0923 [M+H].

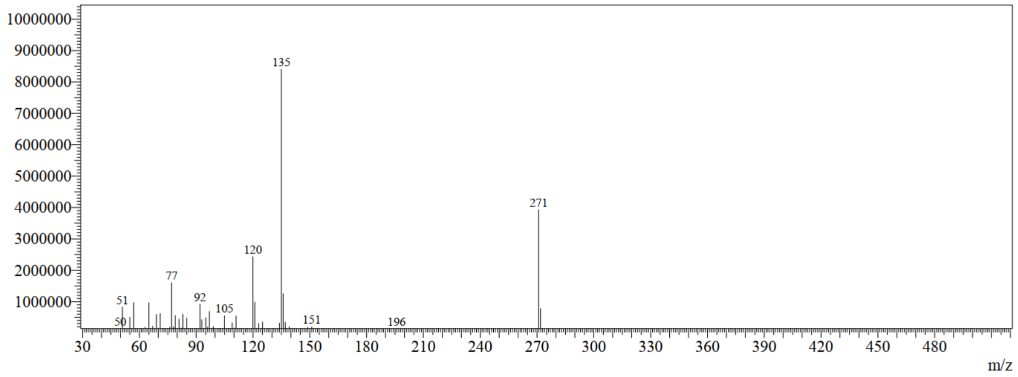

Figure S10. GC-MS spectrum of compound 4

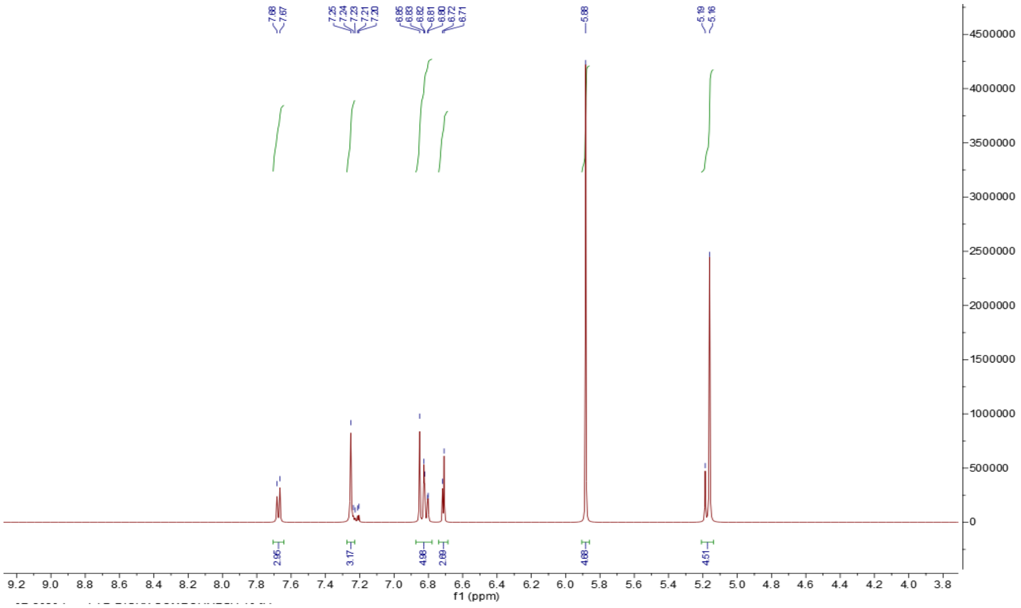

Figure S11. ^{1}H NMR spectrum of compound 4

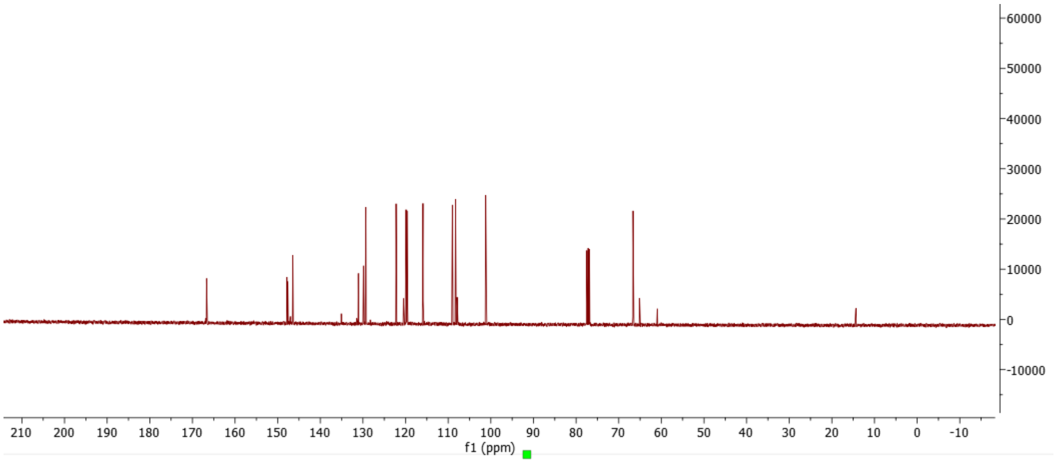

Figure S12. ^{13}C NMR spectrum of compound 4

Benzo[d][1,3]dioxol-5-ylmethyl 4-fluorobenzoate **(5):** white solid; IR (KBr) (ν_{max}/cm^{-1}): 1706, 1502, 1441, 1250, and 1409 cm^{-1}. ^{1}H NMR (400 MHz, $CDCl_3$). δ = 5.18 (s, 2 H), 5.91 (s, 2 H), 6.73 (d, 1 H), 6.86-6.87 (d, 1 H), 7.03 (s, 1 H), 7.98-8.00 (d, 2 H), 8.00-8.02 (d, 2 H). ^{13}C NMR (100 MHz, $CDCl_3$): δ = 66.85, 101.23, 107.88, 108.29, 109.06, 115.55, 122.42, 129.64, 132.29, 147.04, 147.88, 164.54, 165.47. MS (ESI): m/z calcd for $C_{15}H_{11}FO_4$+Na:297.05; found: 297.0538 [M+Na].

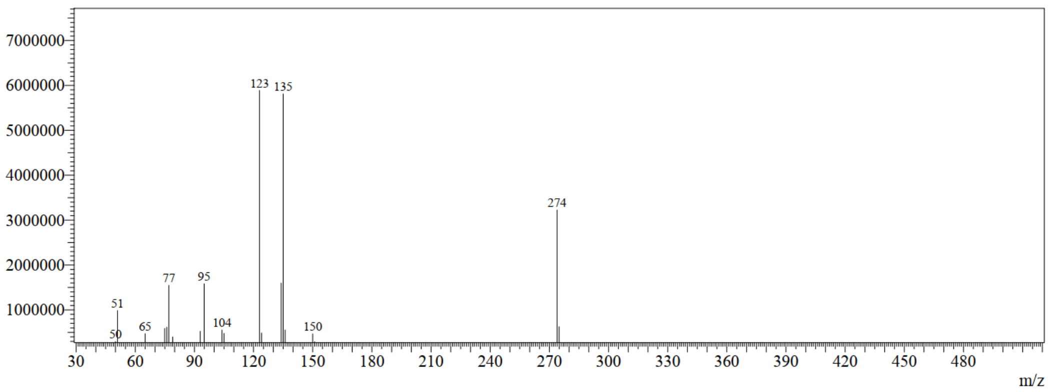

Figure S13. GC-MS spectrum of compound 5

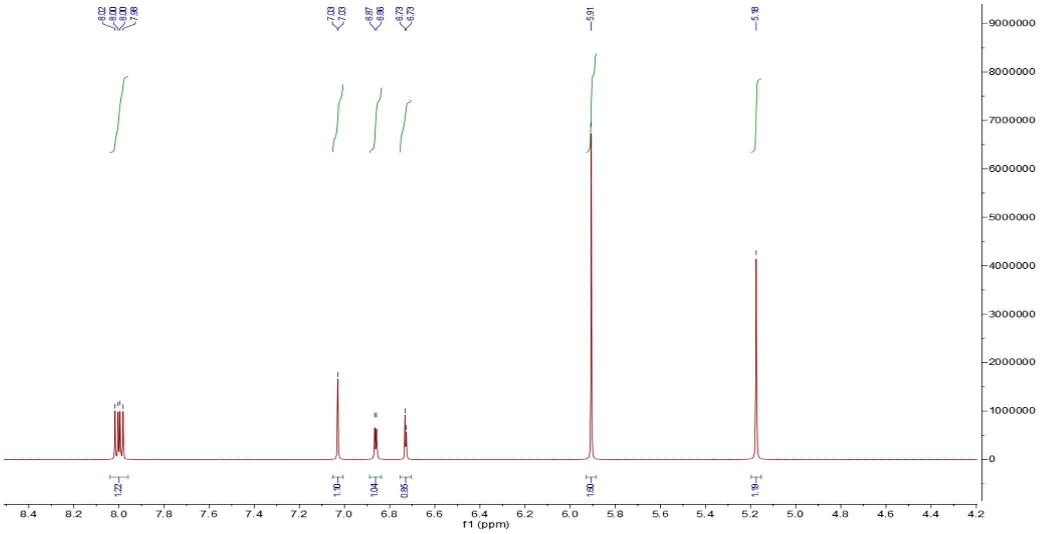

Figure S14. ^{1}H NMR spectrum of compound 5

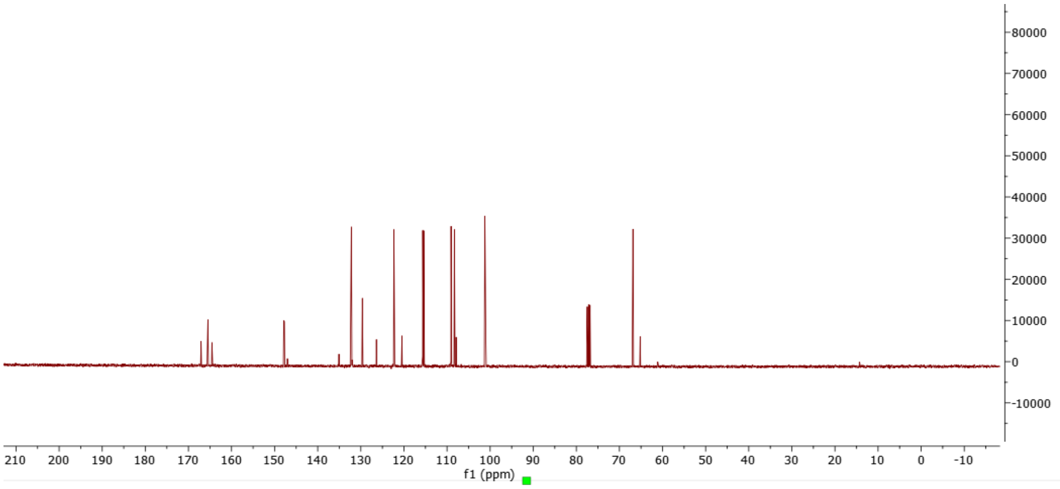

Figure S15. ^{13}C NMR spectrum of compound 5

***Benzo[d][1,3]dioxol-5-ylmethyl 2,2,2-trifluoroacetate* (6):** white solid; IR (KBr) (ν_{max}/cm^{-1}): 1602, 1487, 1269, and 1368 cm^{-1}. ^{1}H NMR (400 MHz, $CDCl_3$). δ = 4.52 (s, 2 H), 5.89 (s, 2 H), 6.74 (d, 1 H), 6.76 (d, 1 H), 6.80 (s, 1 H). ^{13}C NMR (100 MHz, $CDCl_3$): δ = 64.93, 101, 106.94, 107.89, 108.37, 120.48, 134.93, 146.97, 147.73. MS (ESI): m/z calcd for $C_{10}H_7F_3O_4$+H:249.16; found: 249.853 [M+H].

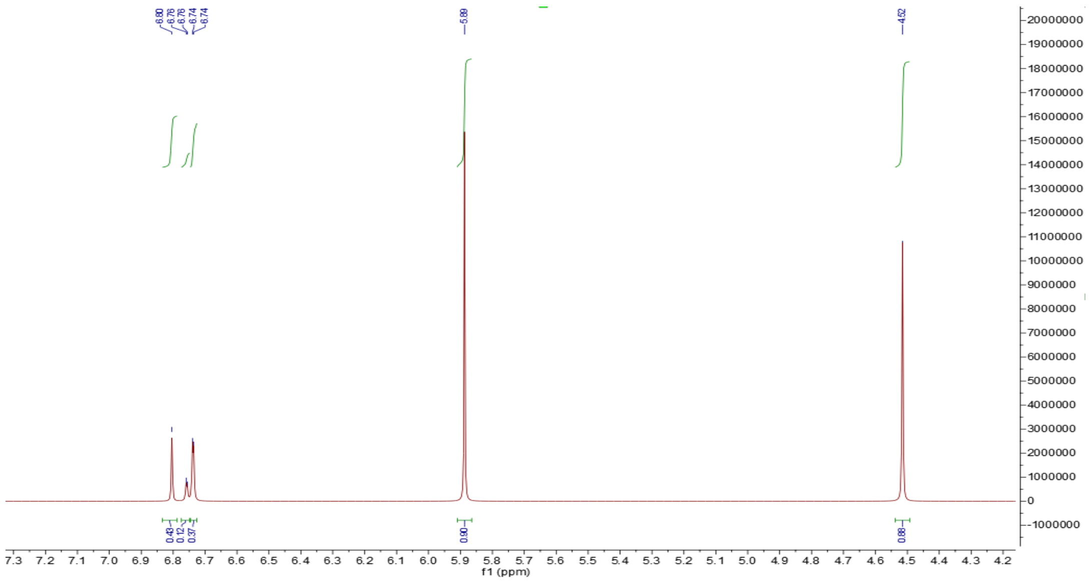

Figure S16. ^{1}H NMR spectrum of compound 6

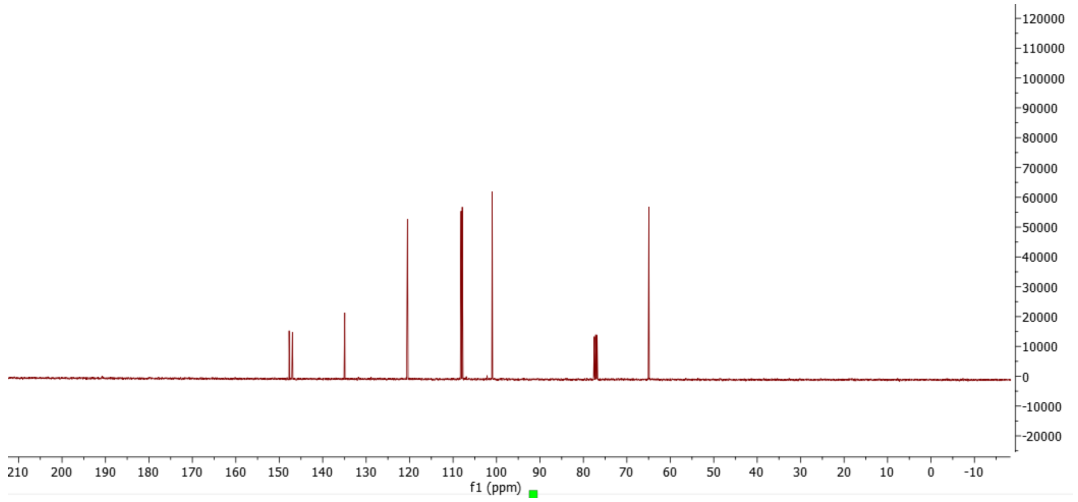

Figure S17. ^{13}C NMR spectrum of compound 6

***Benzo[d][1,3]dioxol-5-ylmethyl benzoate* (7):** white solid; IR (KBr) (ν_{max}/cm^{-1}): 1718, 1495, 1443, and 1257. ^{1}H NMR (400 MHz, $CDCl_3$). δ = 5.17 (s, 2 H), 5.86 (s, 2 H), 6.79 (d, 1 H), 6.83 (d, 1 H), 6.86 (s, 1 H), 7.31-7.35 (m, 2 H), 7.44-7.48 (m, 1 H), 7.97 (d, 2 H). ^{13}C NMR (100 MHz,

CDCl3): δ = 66.73, 101.25, 108.11, 109.07, 122.12, 128.46, 129.65, 130.17, 133.01, 147.39, 147.92, 166.41. MS (ESI): m/z calcd for $C_{15}H_{12}O_4$+Na:279.06; found: 279.0632 [M+Na].

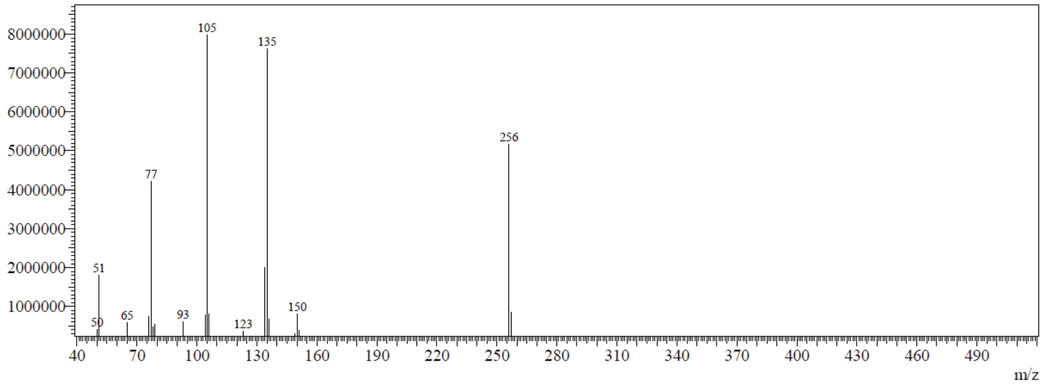

Figure S18. GC-MS spectrum of compound 7

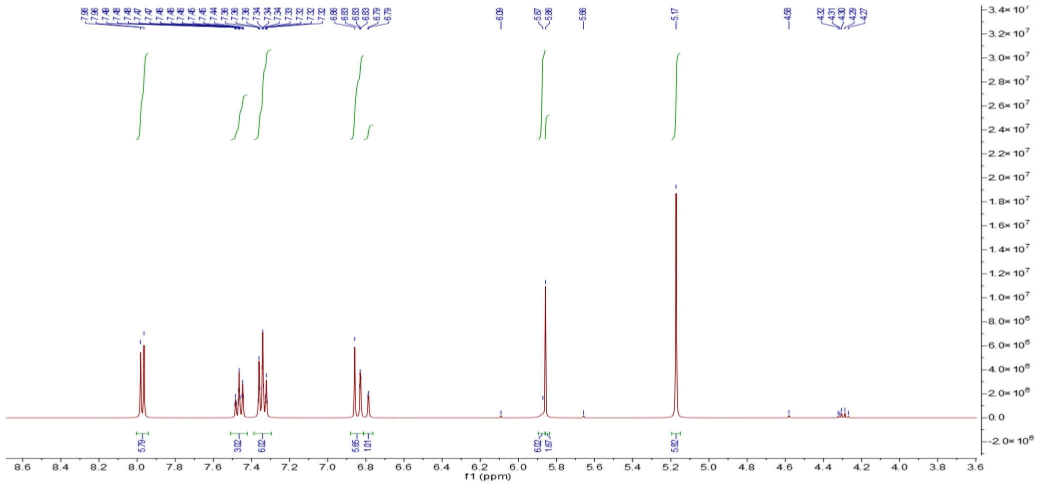

Figure S19. ^{1}H NMR spectrum of compound 7

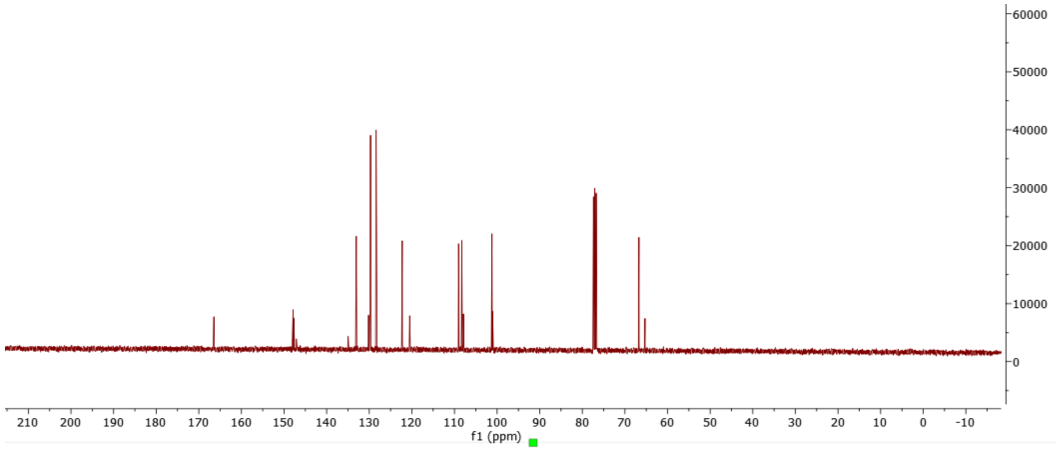

Figure S20. ^{13}C NMR spectrum of compound 7

***Benzo[d][1,3]dioxol-5-ylmethyl 4-methylbenzoate* (8):** white solid; IR (KBr) (ν_{max}/cm^{-1}): 1710, 1481, 1446, 1265, and 1452 cm^{-1}. ^1H NMR (400 MHz, $CDCl_3$). δ = 2.31 (s, 3 H), 5.14 (s, 2 H), 5.85 (s, 2 H), 6.70 (d, 1 H), 6.81 (d, 1 H), 6.84 (s, 1 H), 7.14 (d, 2 H), 7.86 (d, 2 H). ^{13}C NMR (100 MHz, $CDCl_3$): δ = 21.63, 66.43, 101.32, 108.11, 109.07, 122.20, 127.34, 128.98, 129.73, 130.17, 143.74, 147.54, 147.99, 166.48. MS (ESI): m/z calcd for $C_{16}H_{14}O_4$+Na:293.08; found: 293.0788 [M+Na].

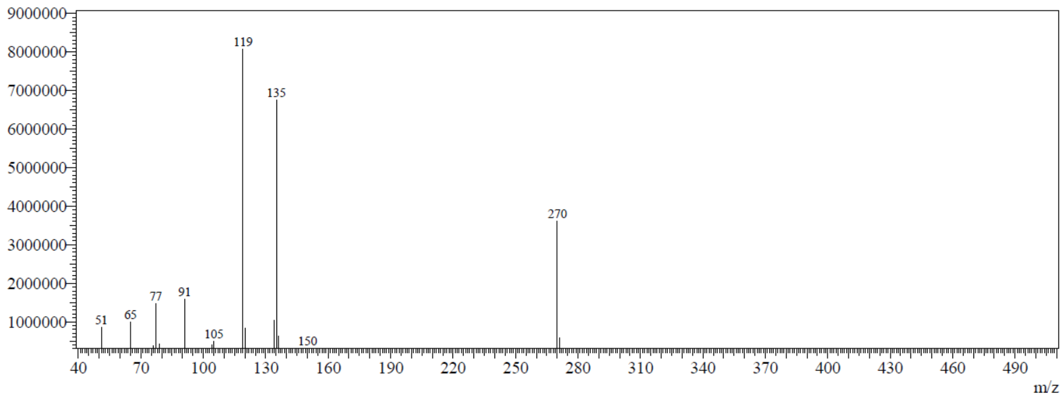

Figure S21. GC-MS spectrum of compound 8

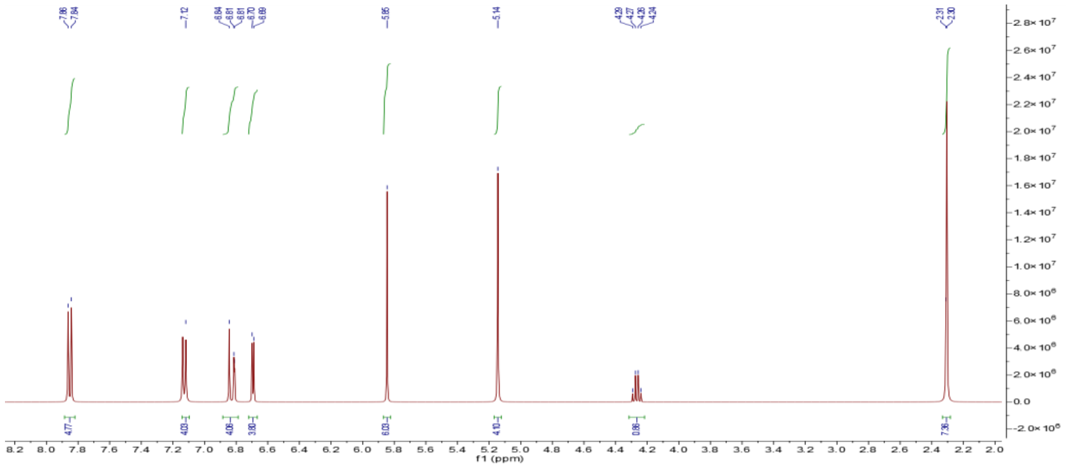

Figure S22. ^1H NMR spectrum of compound 8

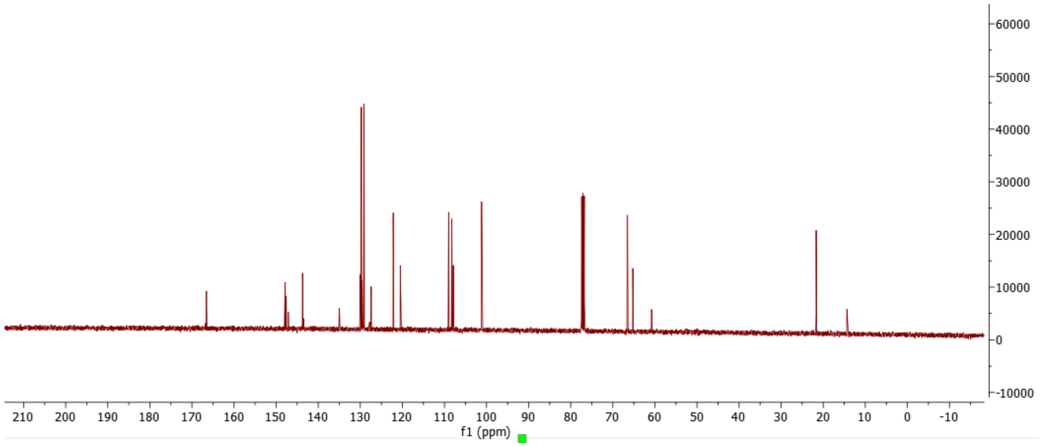

Figure S23. ^{13}C NMR spectrum of compound 8

Synthesis of compound 9. The reactions referred to [29] with modification. Compound **3** (0.6 mmol) was reacted with diphenyl diselenide (0.3 mmol) in 1 mL DMF. Cu_2O (5 mol%), bpy (10 mol%), and Mg (0.6 mmol) were added to the reaction mixture. The mixture was stirred at 110 °C for 24 hours. The reaction was monitored by TLC and GC-MS. After the completion reaction, the mixture was cooled to room temperature. Then, crude product was separated by extraction process using dichloromethane and brine solution. The organic layer was evaporate and the crude product was purified by column chromatography over silica gel.

benzo[d][1,3]dioxol-5-ylmethyl 4-(phenylselanyl)benzoate **(9):** yellow liquid; IR (KBr) (ν_{max}/cm^{-1}): 1723, 1436, 1383, 1254, and 1088 cm^{-1}. ^{1}H NMR (400 MHz, $CDCl_3$). δ = 5.97 (s, 2 H), 6.92 (s, 1 H), 6.89 (d, 1 H), 6.80 (d, 1 H), 5.24 (s, 2 H), 7.88 (d, 1 H), 7.78 (d, 1 H), 7.37 (d, 2 H), 7.61 (dd, 2 H), 7.76 (dd, 1 H). ^{13}C NMR (100 MHz, $CDCl_3$): δ = 65.15, 101.01, 106.93, 108.38, 122.01, 127.75, 128.71, 129.21, 131.52, 133.54, 135.10, 148.74, 153.14, 190.33. HRMS (ESI): m/z calcd for $C_{21}H_{16}O_4$Se+Na:434.29; found: 434.0937 [M+Na].

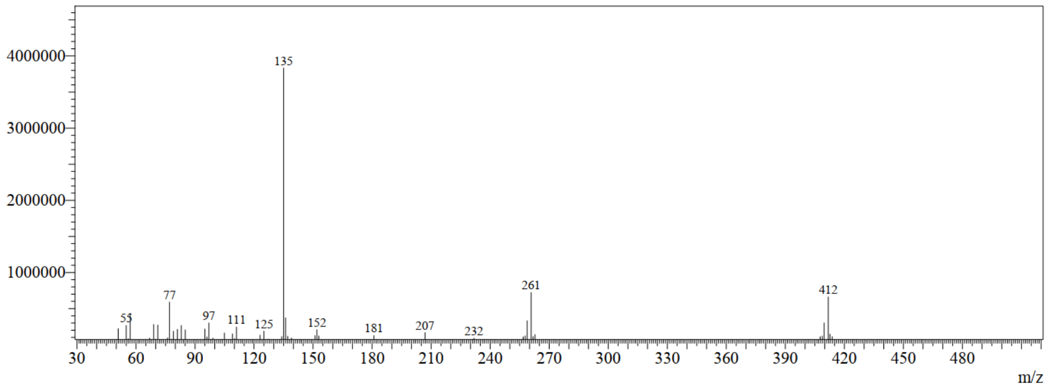

Figure S24. GC-MS spectrum of compound 9

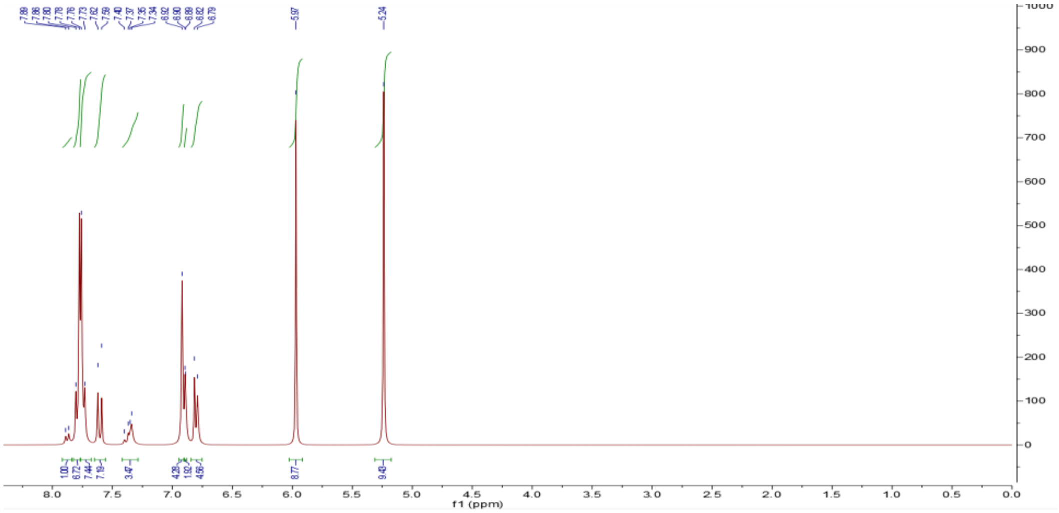

Figure S25. ^{1}H NMR spectrum of compound 9

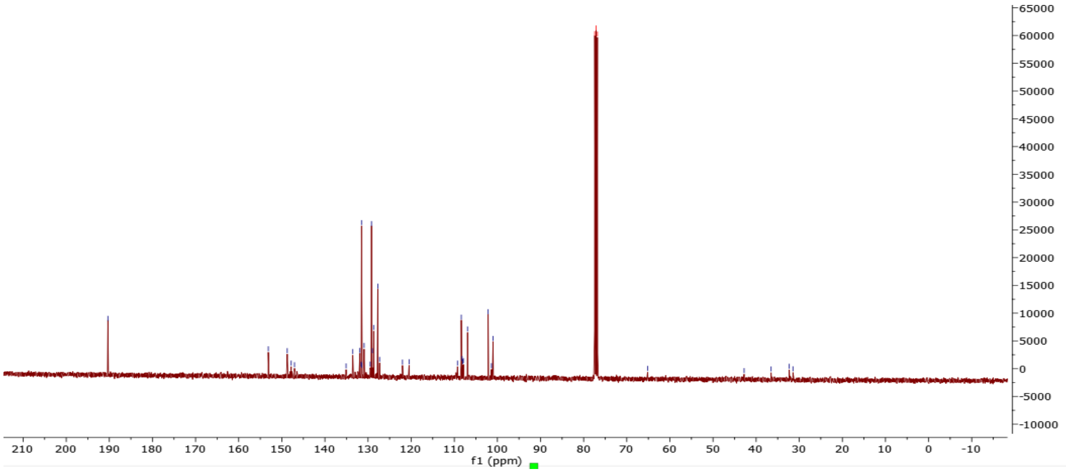

Figure S26. ^{13}C NMR spectrum of compound 9

Synthesis of compound 10. The reactions referred to [30] with modification. Trifluoromethyl phenyl boronic acid **10a** (1.3 mmol) was reacted with 2,2-dithiobis(benzothiazole) **10b** (0.6 mmol) in DMSO:water (2:1). CuI;bpy (10 mol%) was added to the reaction mixture. The mixture was stirred at 110 °C for 3 hours. The reaction was monitored by TLC and GC-MS. After the completion reaction, the mixture was cooled to room temperature. Then, crude product was separated by extraction process using dichloromethane and brine solution. The organic layer was evaporate and the crude product was purified by column chromatography over silica gel.

***2-((4-(trifluoromethyl)phenyl)thio)benzo[d]thiazole* (10):** yellow solid; IR (KBr) (ν_{max}/cm^{-1}): 2110, 1465, 1404, 1308, and 1310 cm^{-1}. ^{1}H NMR (400 MHz, $CDCl_3$). δ = 7.38 (d, 2 H), 7.55 (d, 2 H), 7.76-7.80 (dd, 1 H), 7.86-7.89 (dd, 1 H), 8.00 (d, 1 H), 8.02 (d, 1 H). ^{13}C NMR (100 MHz, $CDCl_3$): δ = 121.28, 121.32, 122.62, 123.02, 124.64, 125.79, 126.69, 134.18, 136.59, 152.81, 160.02. HRMS (ESI): m/z calcd for $C_{14}H_8F_3NS_2$+H:312.01; found: 312.0129 [M+H].

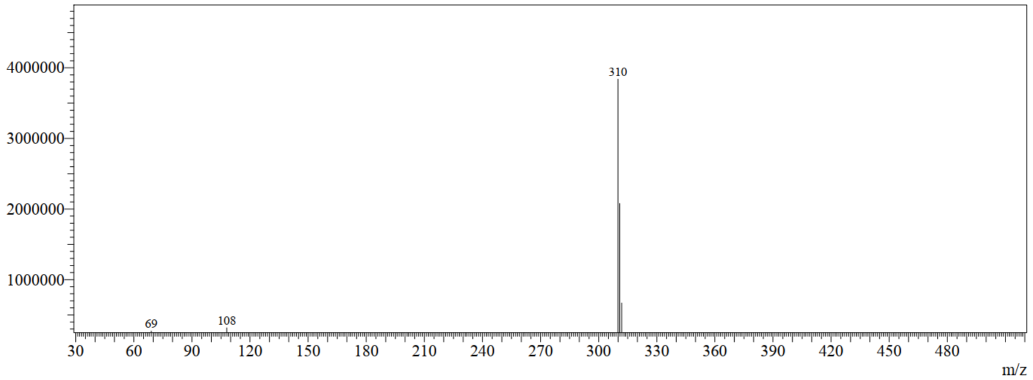

Figure S27. GC-MS spectrum of compound 10

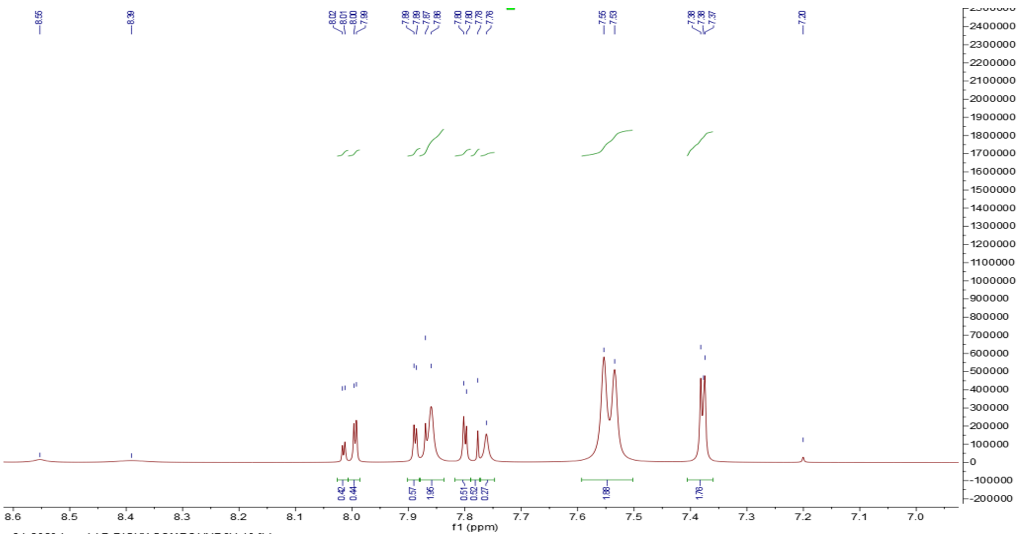

Figure S28. ^{1}H NMR spectrum of compound 10

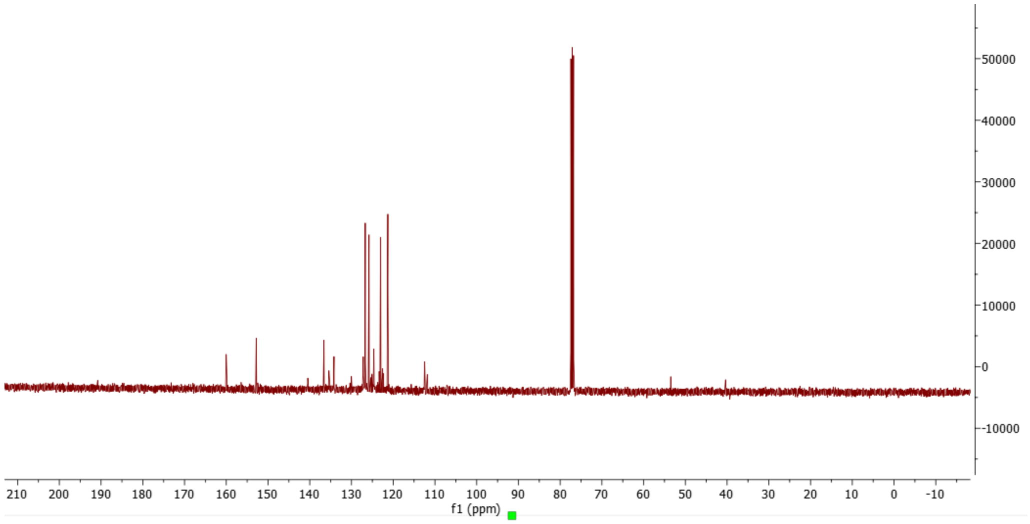

Figure S29. ^{13}C NMR spectrum of compound 10

In vitro LDHA activity assay

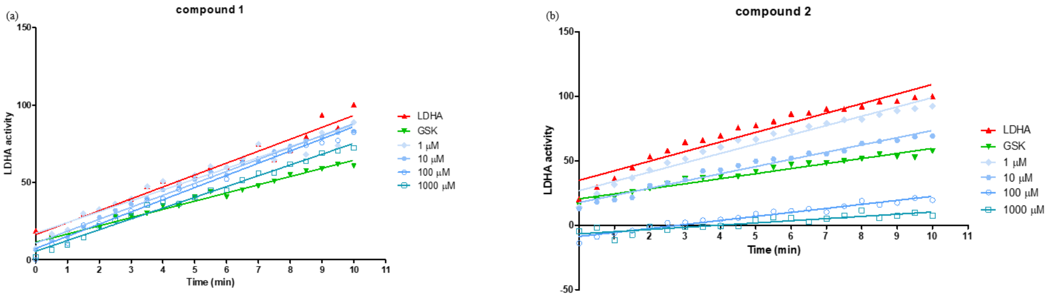

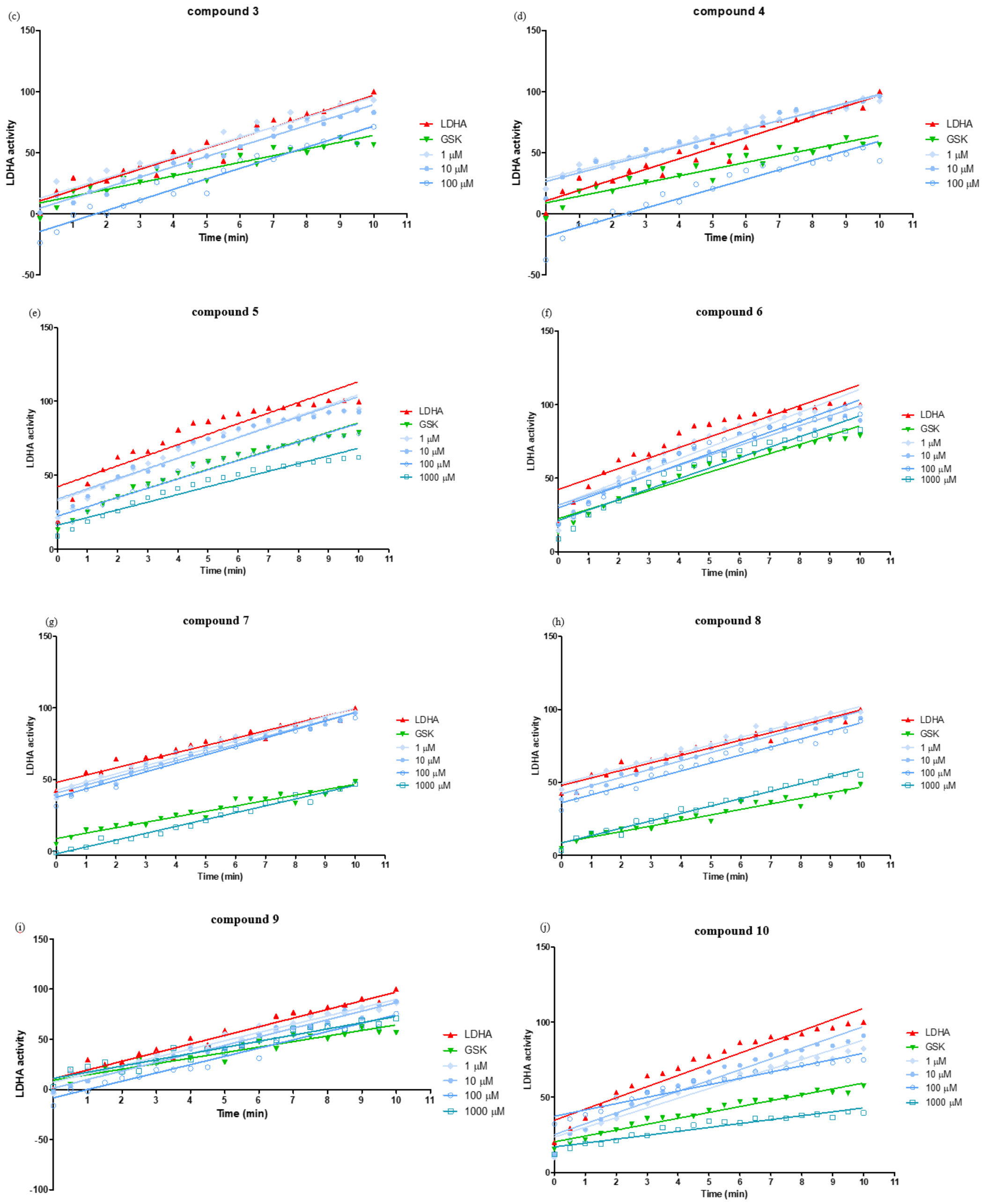

Figure S30. *In vitro* LDHA activity assay of compound 1 to 10 (a-j)

In vitro LDHB activity assay

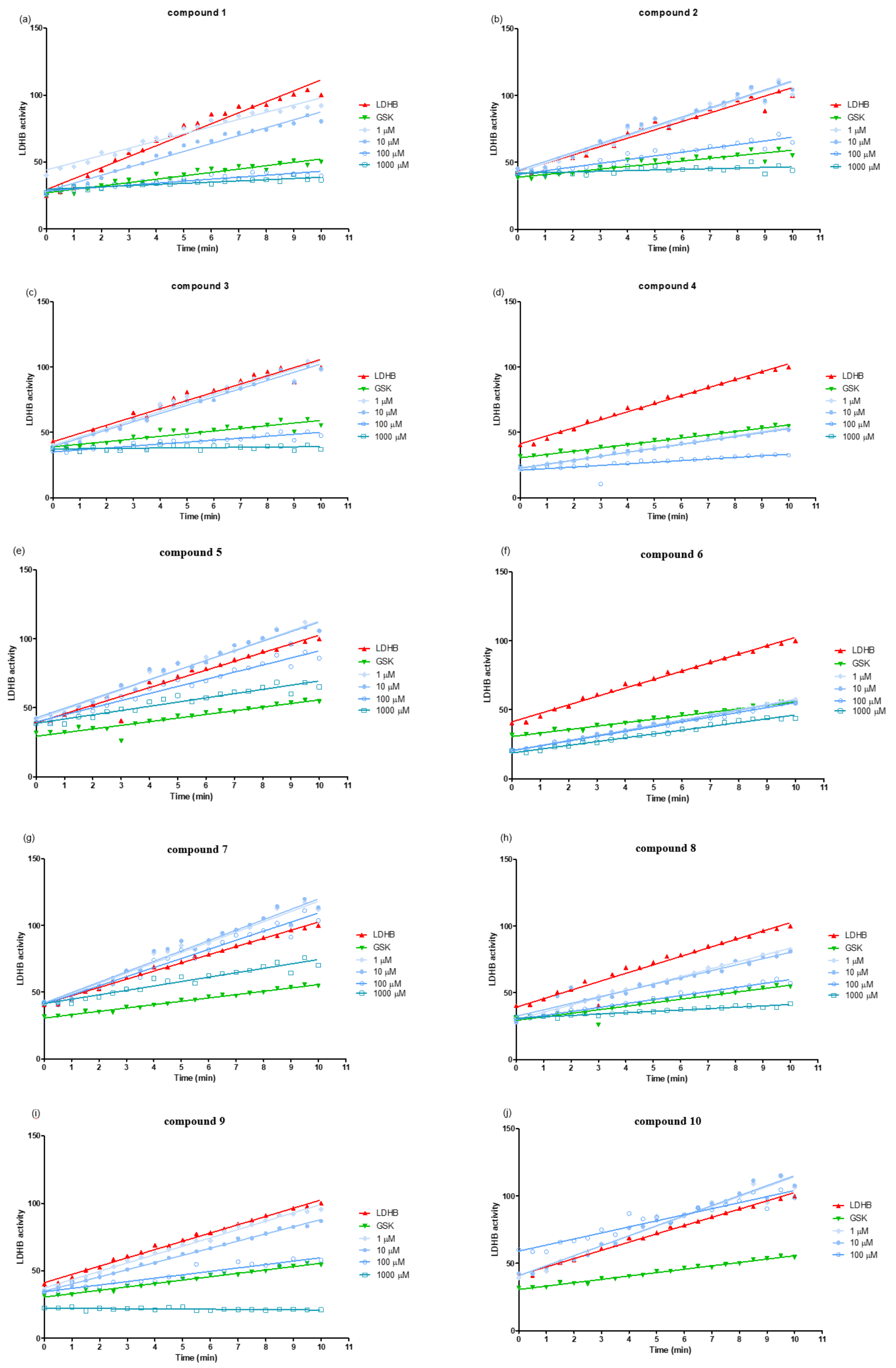

Figure S31. *In vitro* LDHB activity assay of compound 1 to 10 (a-j)

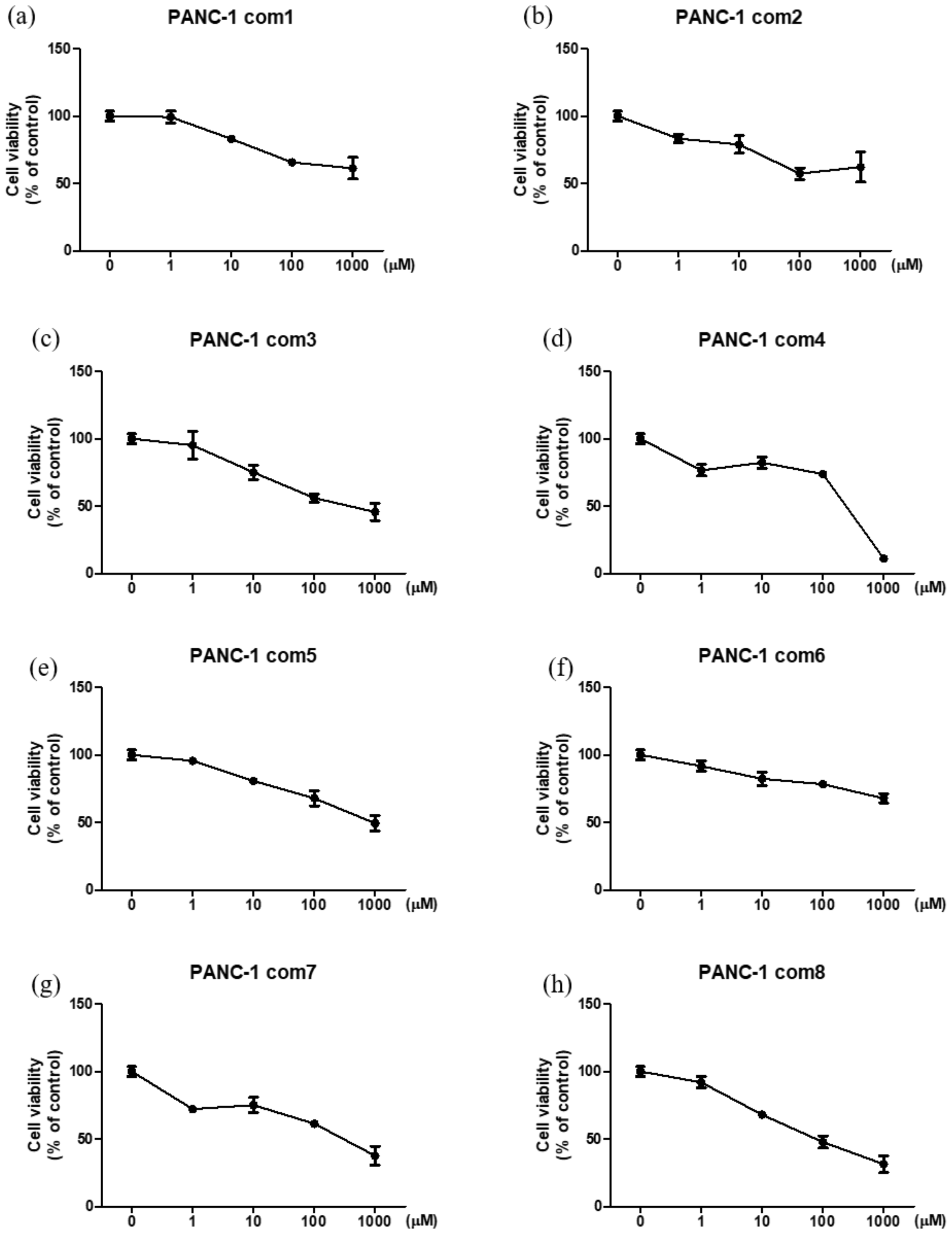
(a)
PANC-1 com1
(b)
PANC-1 com2
(c)
PANC-1 com3
(d)
PANC-1 com4
(e)
PANC-1 com5
(f)
PANC-1 com6
(g)
PANC-1 com7
(h)
PANC-1 com8
Cell viability
(% of control)
150
100
50
0
0
1
10
100
1000
(μM)

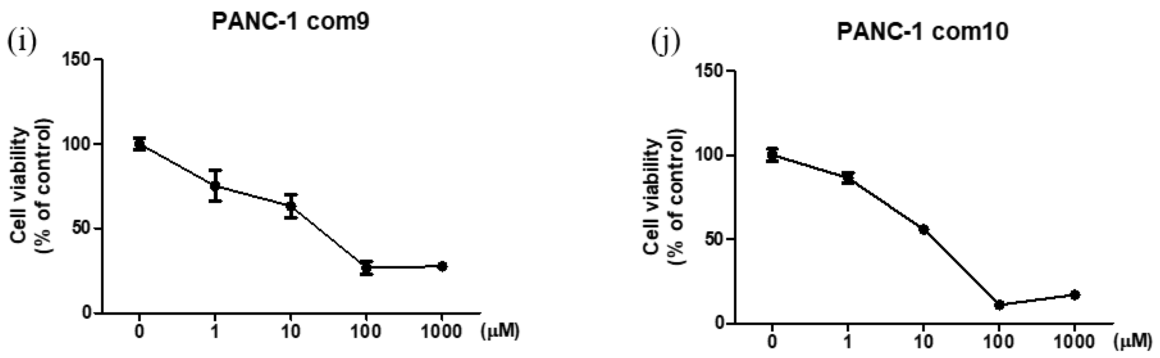

Figure S32. Cellular toxicity assay of compound 1 to 10 in PANC-1 cells (a-j)

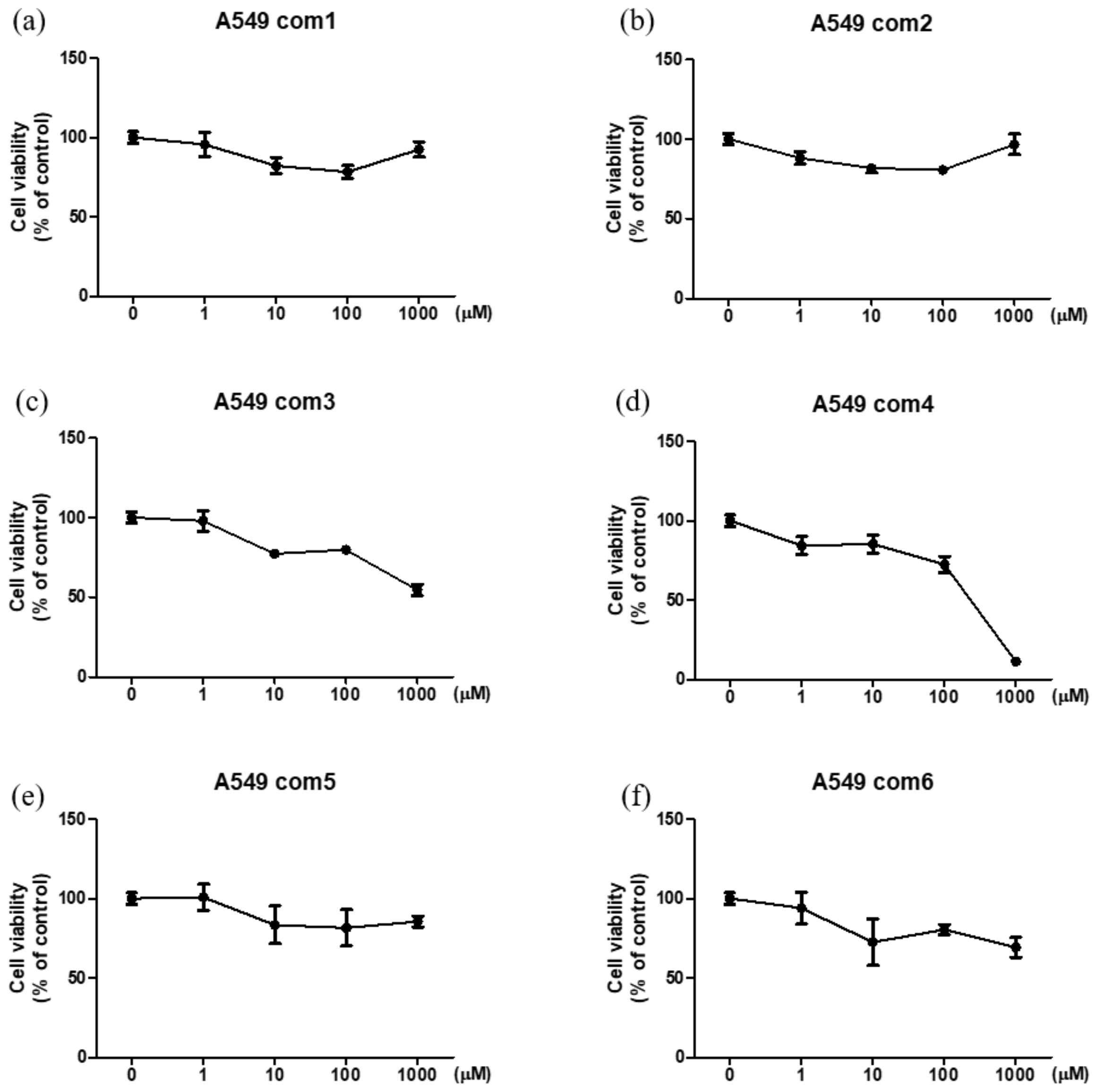

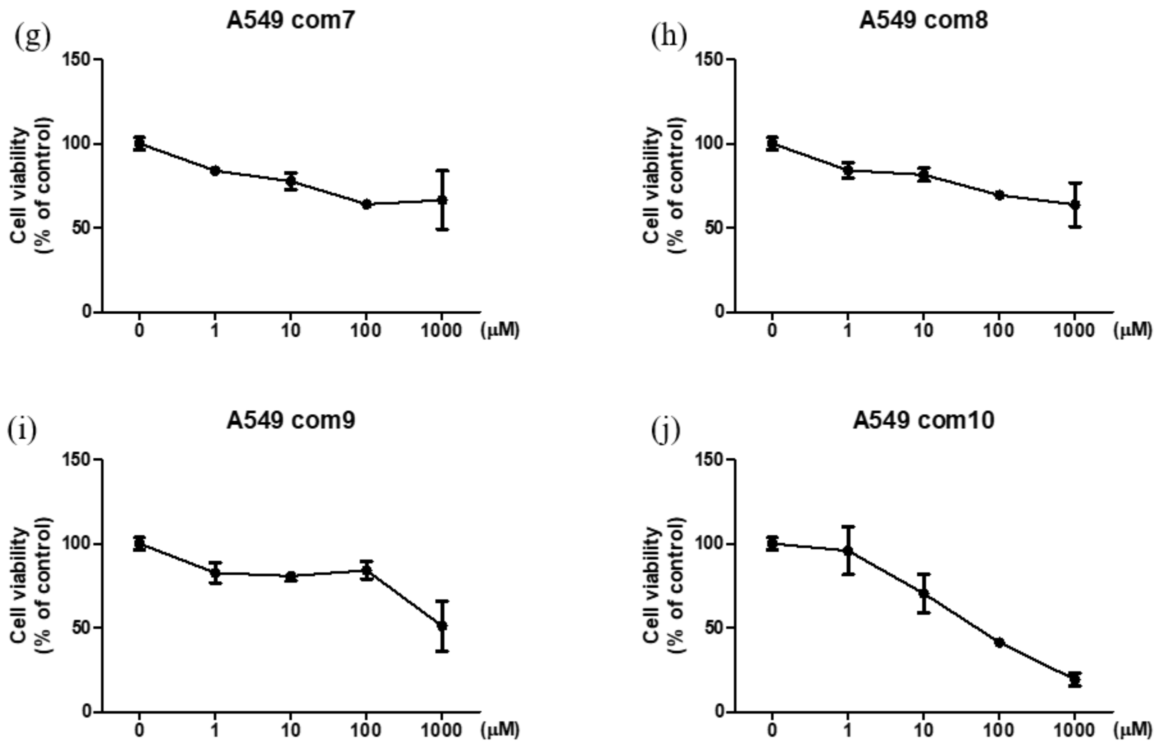

Figure S33. Cellular toxicity assay of compound 1 to 10 in A549 cells (a-j)

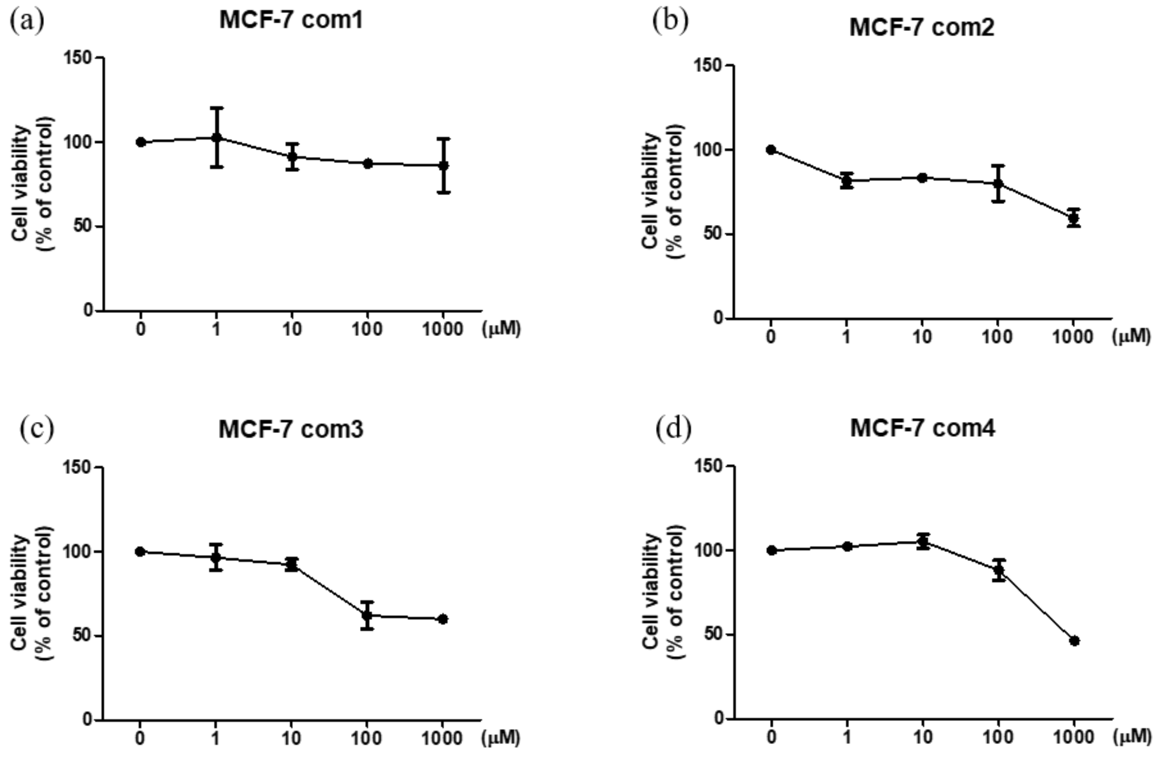

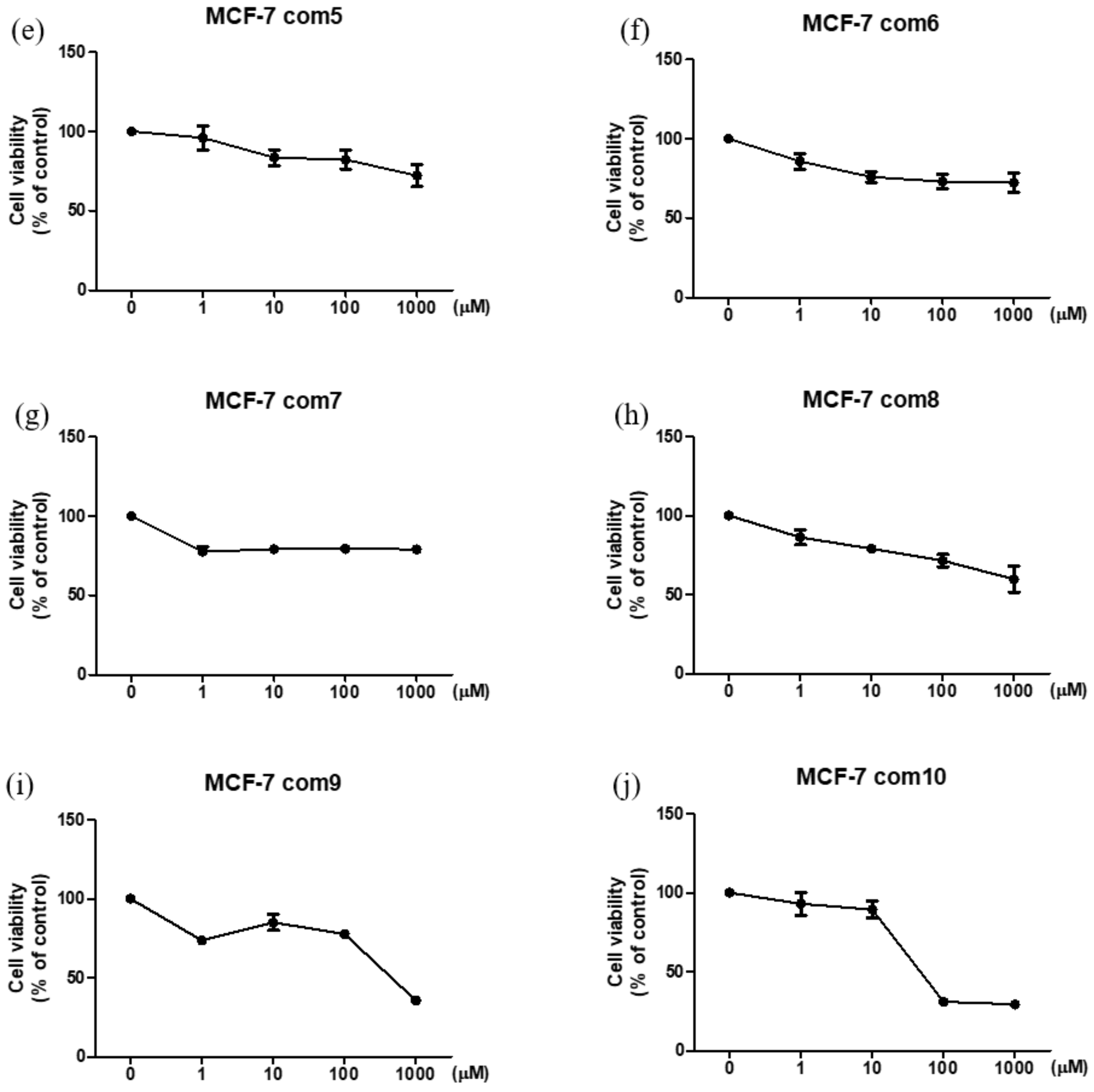

Figure S34. Cellular toxicity assay of compound 1 to 10 in MCF-7 cells (a-j)

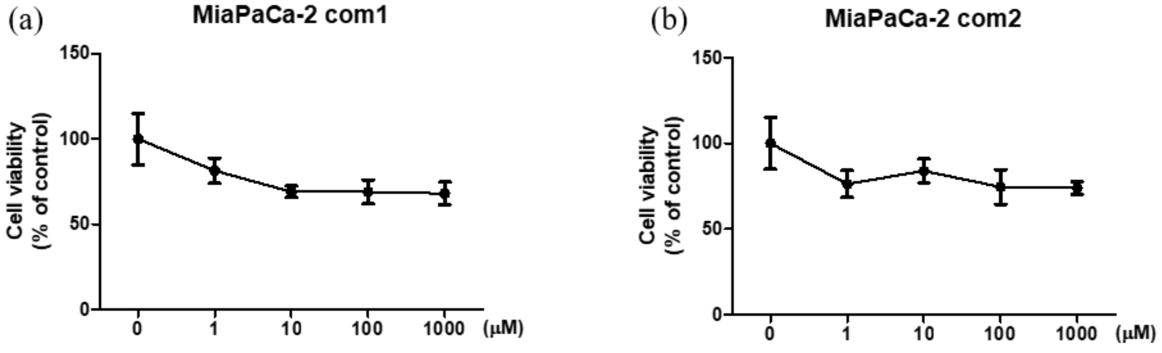

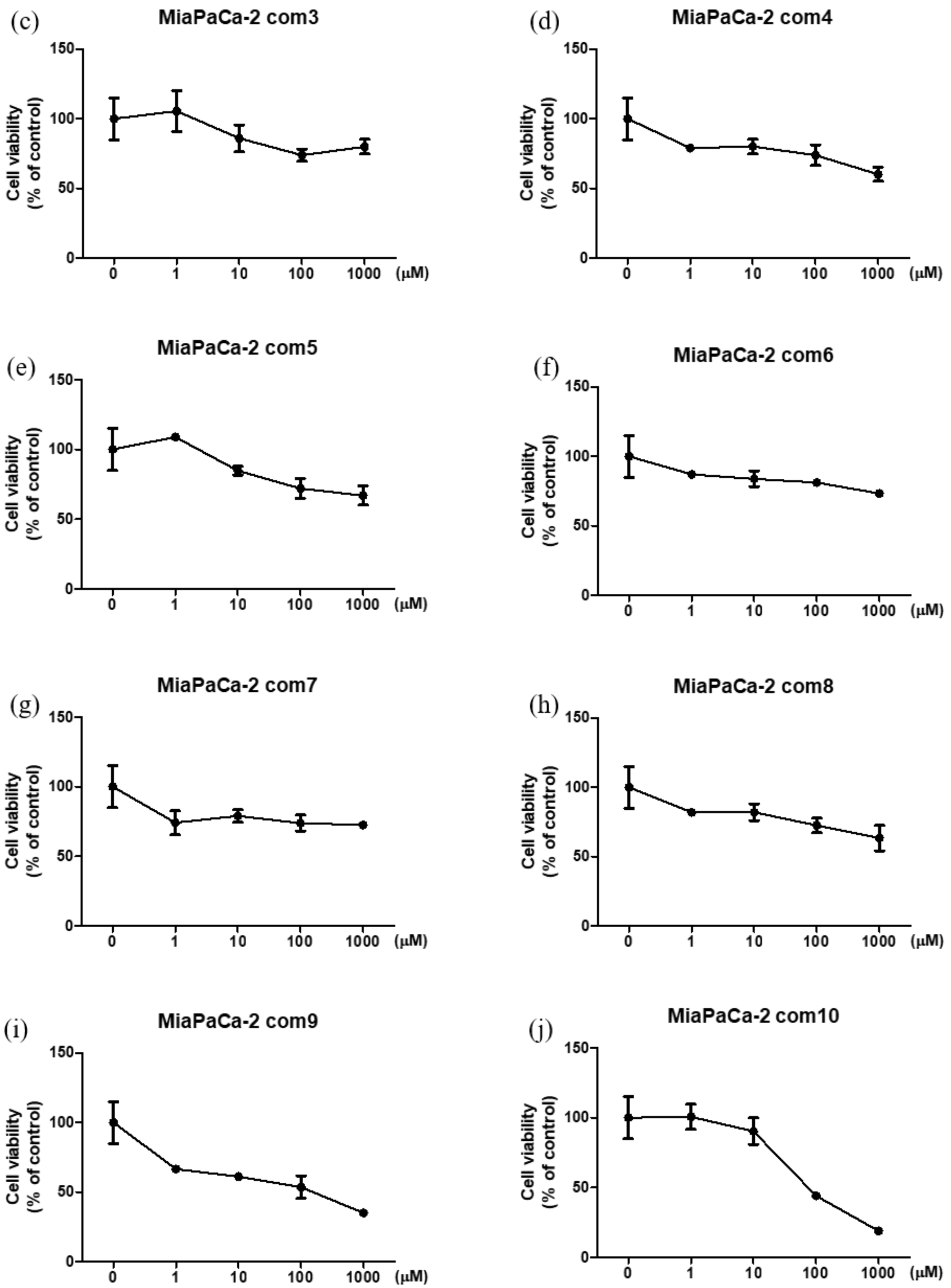

Figure S35. Cellular toxicity assay of compound 1 to 10 in MiaPaCa-2 cells (a-j)

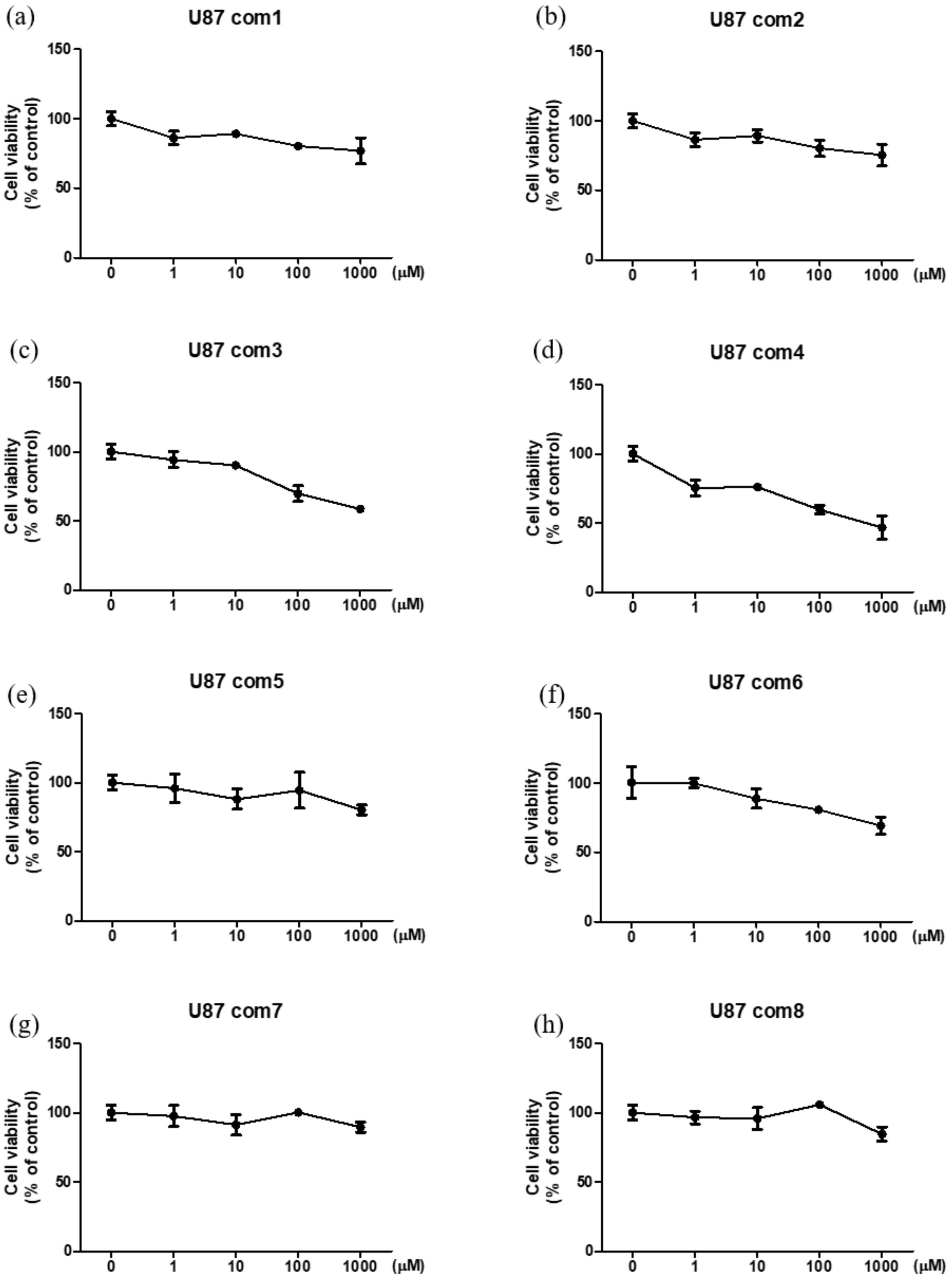
(a)
U87 com1
(b)
U87 com2
(c)
U87 com3
(d)
U87 com4
(e)
U87 com5
(f)
U87 com6
(g)
U87 com7
(h)
U87 com8
Cell viability
(% of control)
150
100
50
0
0
1
10
100
1000
(μM)

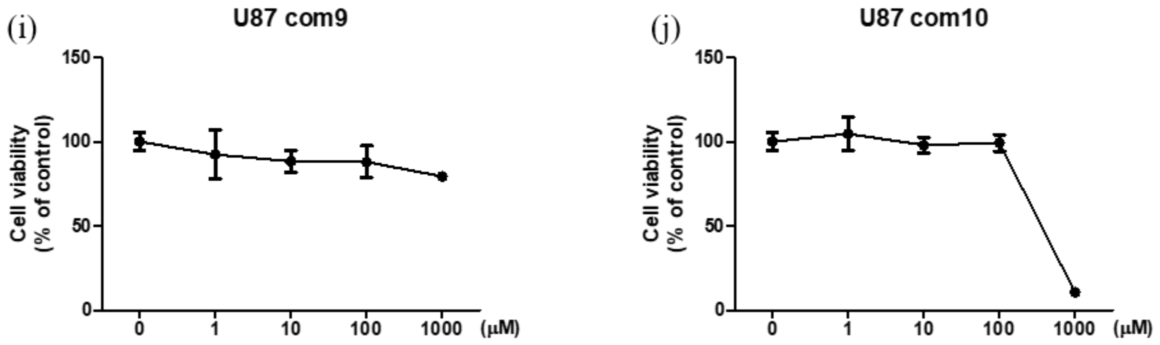

Figure S36. Cellular toxicity assay of compound 1 to 10 in U87 cells (a-j)

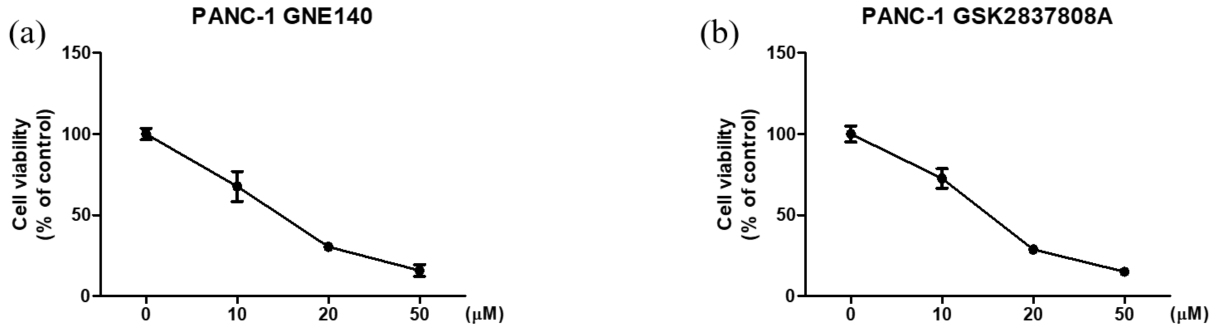

Figure S37. Cellular toxicity assay of GNE140 (a) and GSK2837808A (b) in PANC-1 cells
